# Reproducibility of sublingual microcirculation parameters obtained from sidestream darkfield imaging

**DOI:** 10.1371/journal.pone.0213175

**Published:** 2019-03-14

**Authors:** Luca Valerio, Ron J. Peters, Aeilko H. Zwinderman, Sara-Joan Pinto-Sietsma

**Affiliations:** 1 Department of Public Health, Amsterdam UMC, University of Amsterdam, Amsterdam, Netherlands; 2 Department of Cardiology, Amsterdam UMC, University of Amsterdam, Amsterdam, Netherlands; 3 Department of Clinical Epidemiology and Biostatistics, Amsterdam UMC, University of Amsterdam, Amsterdam, Netherlands; 4 Department of Vascular Medicine, Amsterdam UMC, University of Amsterdam, Amsterdam, Netherlands; University Medical Center Utrecht, NETHERLANDS

## Abstract

**Background:**

Changes in the microcirculation may be used as a surrogate outcome in studies on cardiovascular disease. We assessed the reliability characteristics of the sublingual microcirculation parameters Vascular Density (VD), Red Blood Cell Filling (RBCF), and Perfused Boundary Region (PBR) as obtained by sidestream darkfield imaging.

**Methods:**

For each of the three parameters, the variance components of measurement, the Intraclass Correlation Coefficient (ICC), the Standard Error of Measurement, and the limits of agreement were estimated for the intra-rater setting (N = 50) and the inter-rater setting (N = 48). Subsequently, as a proof of concept, the reliability measures were used for a power analysis to design studies to evaluate the effect of acute stimuli–i.e. having a meal (N = 50) and cigarette smoking (N = 21) on the three parameters.

**Results:**

Reproducibility was poor for all three parameters. The intra-rater ICC for 2 measurements was 0.28 (95% CI: 0.04, 0.53) for the VD, 0.51 (95% CI: 0.27, 0.69) for the RBCF, and 0.33 (95% CI: 0.08–0.56) for the PBR. The standard errors of measurement and the limits of agreement for all three parameters were larger than most statistically significant intra-individual or inter-individual differences reported in previous studies. The proofs of concept showed that sample sizes in excess of 600 subjects are necessary to reach statistical significance for the observed effects of having a meal or smoking on VD and PBR.

**Conclusions:**

The reliability of the three sublingual microcirculation parameters in their current form appears to be low and a large sample size is advisable for their use in conditions similar to those we describe.

## Introduction

Longitudinal investigation of cardiovascular disease is time-expensive and requires large groups of participants. In the search for reliable surrogate markers of disease, microcirculation has been proposed as a candidate target. For this purpose, the Sidestream Dark Field [1] video recordings are increasingly used to measure the sublingual microcirculation in the general population, after measurements conducted using its precursor, Orthogonal Polarization Spectral imaging, had been suggested to be of prognostic relevance in critically ill patient populations [2]. The parameters used include two markers of microvascular perfusion, the Vascular Density (VD) [3–5] and the Red Blood Cell Filling percentage (RBCF) [5], and an indicator of the endothelial surface layer usually referred to as the glycocalyx, the Perfused Boundary Region (PBR) [6]. Since they can be obtained from a non-invasive, fast, and semi-automated procedure, these parameters have been considered markers of vascular damage with potential diagnostic, prognostic, and therapeutic values[4, 7, 8]. However, subsequent studies did not yield consistent results. In particular, the association of these parameters with the risk factors for atherosclerosis has been confirmed by some studies[4, 9] and disputed by others[10]. In order to better determine whether the correlations reported for a biomedical parameter are true or spurious[11], knowing how reliable they are is of paramount importance. In particular, it allows researchers to calculate an adequate sample size for future studies[12] which is necessary to apply this method in clinical practice[13]. We approached this matter in two ways: First, we calculated the contributions of different sources of variation to the total variability of the measurements; second, we calculated parameters of measurement error, which helps to determine the clinical applicability of research findings obtained under comparable conditions [14]. We then performed two experimental proofs of concept to use the reliability parameters for sample size calculation.

## Patients and methods

### Study samples

Our studies were performed in four different samples, totalling 169 individuals. Three of these four samples were drawn from the HELIUS (Healthy Life in an Urban Setting) study, a study on health among six ethnic groups [15]; the fourth sample consisted of healthy volunteers. The first part of our analysis, the reproducibility study, was performed in three samples (two of the HELIUS samples and the healthy volunteers sample) and the second part of our analysis, the experimental proof of concept study, was performed in two samples (the third of the HELIUS samples and the healthy volunteers sample). The clinical features of the participants are reported in Table 1.

**Table 1:** Clinical characteristics of the samples.

Study	Intra-observer, 2 measurements	Intra-observer, 6 measurements	Inter-observer, 2 measurements	Effect of a meal	Effect of smoking
Source	HELIUS	Volunteers	HELIUS	HELIUS	Volunteers
N	50	21	48	50	21
Male sex	24 (48%)	21 (100%)	20 (41.7%)	27 (54%)	21 (100%)
Age, years	42.9 (12.2)	28 (3.3)	44.7 (12.8)	40.1 (12.2)	28 (3.3)
Heart rate, bpm	69.0 (10.1)	-	69.6 (9.0)	68.8 (11.4)	-
Systolic BP, mmHg	128.1 (13.7)	-	129.3 (19.5)	127.3 (15.0)	-
Diastolic BP, mmHg	80.5 (10.9)	-	78.8 (11.9)	76.1 (10.1)	-
Hypertension	19 (38%)	0 (0%)	17 (35.4%)	11 (22%)	0 (0%)
BMI, kg/m^2^	28.6 (4.7)	-	27.8 (4.4)	28.2 (4.8)	-
Diabetes	4 (8%)	0 (0%)	6 (12.5%)	6 (12%)	0 (0%)
Current smoker	11 (22%)	0 (0%)	6 (12.5%)	11 (22%)	0 (0%)
CPAD	1 (2%)	0 (0%)	2 (4.2%)	3 (6%)	0 (0%)

Categorical variables are reported as N (%), normal continuous variables as mean (SD).

BP = Blood Pressure. CPAD = Coronary or Peripheral Artery Disease. Bpm = beats per minute.

Hypertension was defined as use of antihypertensive drugs, measured systolic blood pressure ≥ 140 mmHg, or measured diastolic blood pressure ≥ 90 mmHg.

Coronary or Peripheral Artery Disease was defined as self-reported history of myocardial infarction or of coronary or peripheral bypass or revascularization procedure.

The proportion of missing clinical variables in each sample was lower than 10% for each variable.

### Reproducibility studies

The reproducibility studies consisted of an intra-rater reliability analysis and an inter-rater reliability analysis. Intra-rater reliability was investigated in 2 separate studies: the first used 2 measurements spaced 1 minute apart (N = 50, 48% male, mean age 42.9 ±12.2 years), the second used 6 measurements spaced 3 minutes apart (N = 21, 100% male, mean age 28±3.3 years). Inter-rater reliability was investigated in one study, in which five different raters performed the measurements. This was done in four groups of 12 subjects each (N = 48, 42% male, mean age 44.7±12.8 years). In each group, each subject was measured twice, by the first rater and by one of the other four raters; the second measurement began one minute after the end of the first measurement, and between the two measurements, the participant remained seated and was not allowed to stand up. The two measurements were then compared.

### Proof of concept studies

Two additional analyses were performed as proofs of concept for the use of the reproducibility parameters in determining the sample size necessary to reach statistical significance in a clinically relevant hypothesis. The endothelial glycocalyx, one of the parameters for whose assessment the microvascular circulation parameters are used, has been found to react to acute stimuli when investigated using other techniques [16, 17]. Because other stimuli known to acutely affect systemic endothelial function include eating a meal [18] and smoking cigarettes [19, 20], we designed two proof of concept studies focusing on the effect of these two stimuli on the sublingual microcirculation. In the first study, the effect of a standard meal on the parameters was studied in 50 participants (54% male, mean age 40.4 ±12.3 years).The first measurement was carried out between 8:00 and 10:00 after participants had been fasting for 8 to 12 hours and undergone one hour of standard non-invasive biometrical measurements in the setting of the HELIUS study. They then underwent blood withdrawal and consumed a standard meal including 200 mL orange juice (84 kcal), 30 g cream cheese crackers (145 kcal) and one Dutch *krentenbol* (sweet bun, 150 kcal), for a total of approximately 390 kcal. The second measurement took place after further 45 minutes of non-invasive physical examination and, therefore, approximately 60 minutes after the first measurement.

In the second study, the effect of cigarette smoking on the microvascular circulation was studied in the sample of 21 male self-reported non-smokers (mean age 28 ±3.3 years), by conducting measurements 5 minutes before and after they smoked two cigarettes marketed as non-light, with all measurement carried out under conditions comparable to those of the HELIUS study (between 8:00 and 10:00, after 8 to 12 hours fasting, on a phlebotomy chair, and using the Standardized Operating Procedure of the HELIUS study; additional information in S2 Appendix) except for being conducted at the University of Amsterdam Academic Medical Centre rather than at a HELIUS study research location and not being preceded by additional biometrical measurements.

### Measurement of the sublingual microvascular parameters

The measurement procedure and the calculation of the sublingual microvascular parameters here have been described in detail elsewhere [5, 6]. In short, the probe of a hand-held sidestream darkfield (SDF) videomicroscope (MicroVision Medical Inc., Wallingford, PA) is placed on the sublingual mucosa of the subject for approximately 2 minutes to obtain video recordings of the sublingual microvasculature. Subsequently, an analysis software (GlycoCheck ICU, Glycocheck BV, Maastricht, the Netherlands) automatically calculates the sublingual microcirculation parameters, such as vascular density (VD), red blood cell filling (RBCF), and perfused boundary region (PBR).

**Fig 1:**
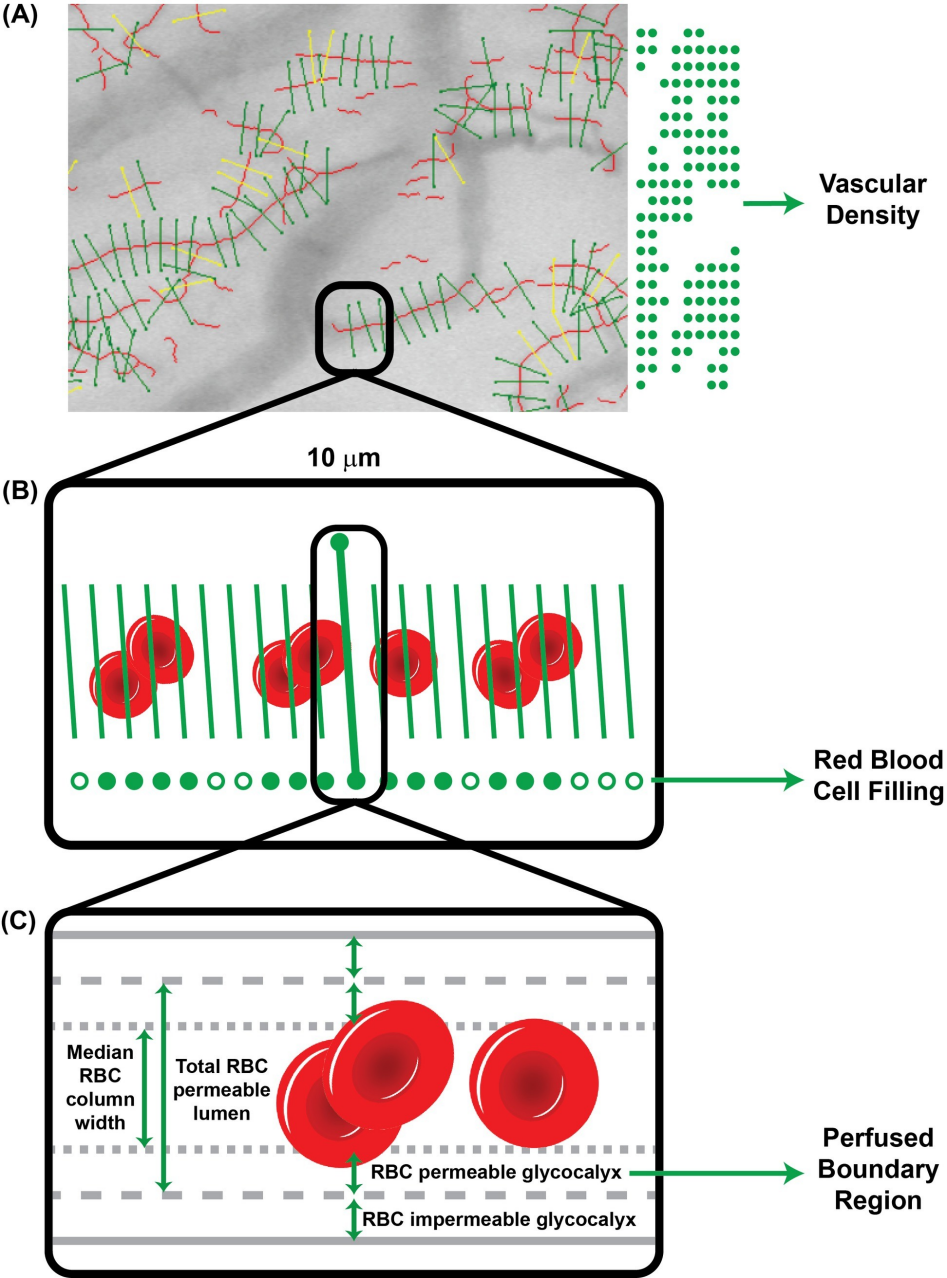
Calculation of Vascular Density, Red Blood Cell Filling, and perfused boundary region from recordings of the sublingual circulation. (A) The Vascular Density is estimated by the total length of the vessels identified in the recorded area. Automatically identified vessels are marked by transversal markers placed at intervals of 10 μm longitudinally along the course of the vessel; each marker therefore contributes 10 μm to the total length. (B) The Red Blood Cell Filling is the proportion of 21 markers (the above main marker and 20 micromarkers placed at 0.5 μm intervals, 10 at either side of the main marker) occupied by Red Blood Cells across all frames of the video recording. (C) The Perfused Boundary Region is the width of the superficial component of the endothelial glycocalyx permeable to Red Blood Cells, estimated by collecting the measured widths of the flowing Red Blood Cell column at each main marker over all recording frames, linearly extrapolating the width of the overall RBC permeable lumen from the 50^th^ (median RBC column width) and the 75^th^ percentiles of the distribution of measured widths, and subtracting the median RBC column width from the width of the overall RBC permeable lumen.

The VD is the length of identified microvessels expressed in μm per square millimeter and calculated from counting the 10 μm long vascular segments identified in each frame. A reduced VD is considered to reflect vascular endothelial damage and an atheroprone risk profile [5]. The RBCF is the proportion in which the red blood cells fill the 10 μm vascular segments. It has also been associated with endothelial damage and an atherogenic risk profile [5]. Finally, the PBR is represented by the lateral movement of the erythrocyte and therefore the space of the vessel that is not occupied by the erythrocyte, assumed to represent the glycocalyx. Measured in μm, an increased PBR reflects increased erythrocyte penetration into the glycocalyx and is considered to be associated with damaged glycocalyx structure or function and, possibly, an atherogenic risk profile[3, 4, 9]. The derivation of all three parameters from a single video recording of the sublingual microcirculation is summarized in Fig 1. Additional information on the data collection in the HELIUS study is available in S2 Appendix and in an excerpt from the internal Standard Operating Procedure of the measurement of sublingual parameters in the HELIUS study.

### Statistical analysis

In all studies, analyses were conducted for each of the three different microvascular parameters and results reported according to the GRRAS guidelines [21].

In the reproducibility studies, the following analyses were performed for both intra-rater reliability studies and the inter-rater reliability studies. First, we attempted to decompose the observed variance of the microvascular parameters into variance components due to true differences between participants, variance due to differences between or within raters and residual variance (due to unknown factors). These variance components and the presence of systematic differences between sequential measurements were studied using a mixed effects linear regression model, in which the sequence of the measurements was a fixed effect, the sources of variation to be attributed to participants a random effect. The higher the variance component due to (true) differences between subjects, the more reliable is the measurement.

Second, two parameters were calculated from this model: the Intraclass Correlation Coefficient (ICC), defined as the proportion of the total observed variance originating from differences between subjects[14], with an ICC value of at least 0.7 considered adequate[22]; and the Standard Error of Measurement (SEM), defined in this study as the square root of the observed residual variance, interpretable as the error to be expected when a measurement is conducted repeatedly on the same subject. Third, a Bland-Altman plot was generated to visually evaluate the presence of interaction between the estimated true value and either the difference between second and first measurement or the spread of observed values around the estimated true value. The smaller the spread and the lower the evidence for interaction, the better the agreement between two measurements. The Bland-Altman plot can explain a low ICC. Lastly, the upper and lower limits of agreement according to Bland-Altman were calculated, which represent the minimal positive or negative difference between repeated measurements in a subject that can be considered to reflect true differences between values with 95% confidence (S1 Fig).

In the proofs of concept, the effect of an acute stimulus on the microvascular parameters was studied using a two-sided t-test for paired samples after visually verifying the parameters were normally distributed. Subsequently, the sample size needed for the observed differences to reach statistical significance was calculated based on the variance components obtained from the intra-rater reliability study [23]. Two-sided *p* values of less than .05 were considered significant. All analyses were performed using R version 3.3.3 (The R Foundation for Statistical Computing, 2017) and packages lme4 version 1.1.17 and lmerTest version 3.0.1; plots were generated using R package ggplot2 version 3.0.0.

### Informed consent

Written informed consent was obtained from all subjects included in the study, the study protocol conformed to the ethical guidelines of the 1975 Declaration of Helsinki and had been priorly approved by the Ethical Review Board of the Academic Medical Centre of the University of Amsterdam (Protocol ID NL32251.018.10, approval number 10/100# 10.17.1729).

## Results

**Fig 2:**
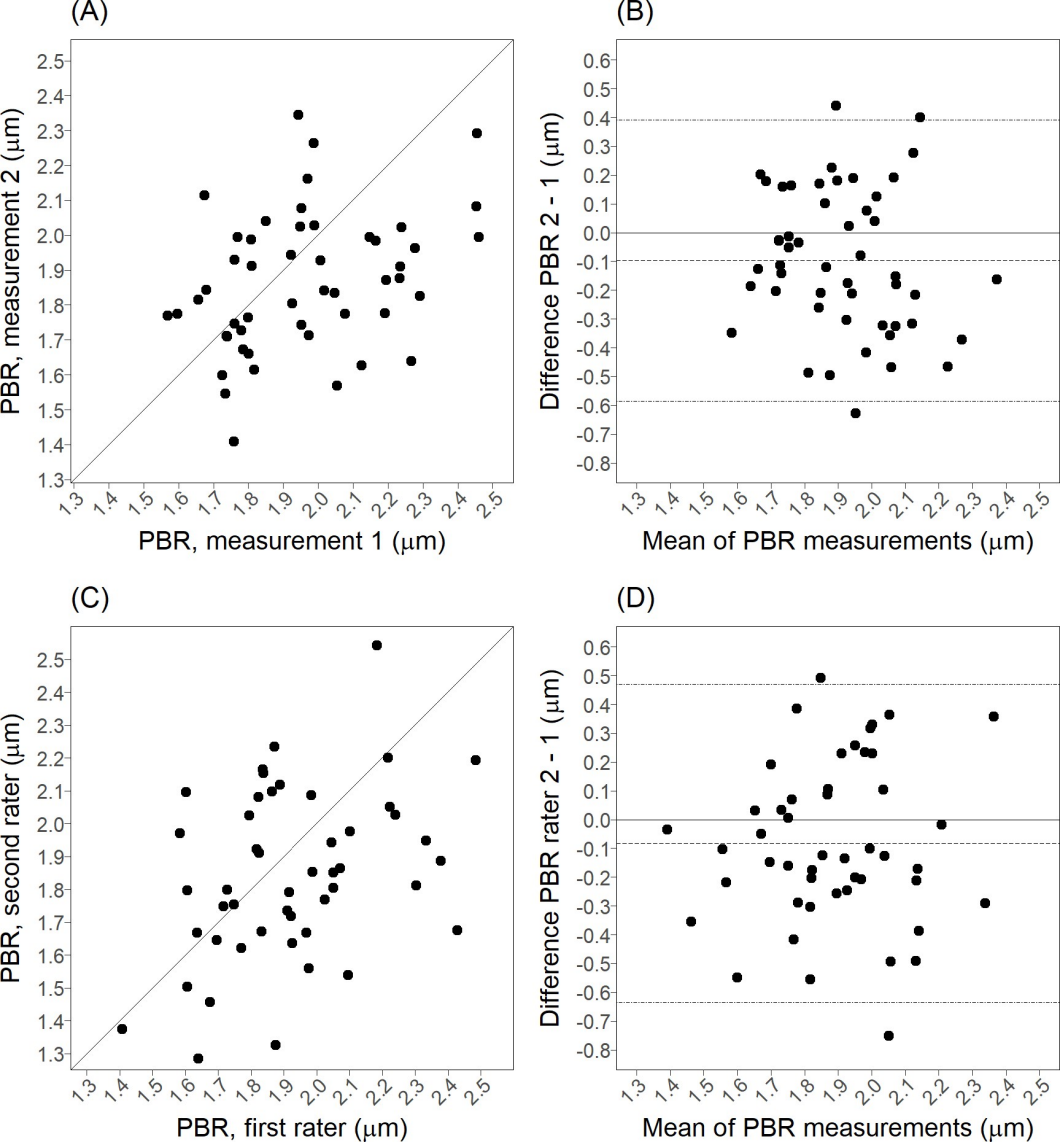
Intra-rater and inter-rater scatter plots and Bland-Altman plots for the PBR. (A) Scatter plot of the intra-rater PBR data. (B) Bland-Altman plot of the intra-rater PBR data. (C) Scatter plot of the inter-rater PBR data. (D) Bland-Altman plot of the inter-rater PBR data.

The results of the reproducibility analyses are shown in Table 2. The Bland-Altman plots for the best researched parameter, the PBR, are shown in Fig 2; those for the RBCF and the VD are shown in S2 Fig.

**Table 2:** Reliability characteristics of the three sublingual microcirculation parameters in the intra-rater and inter-rater studies with two measurements.

Setting	Parameter (unit)	Pearson’s r	ICC (95% CI)	mean difference (95% CI)^b^	P-value	SEM	Limits of agreement
Intra-rater^a^ (1 min. apart)	VD (μm/mm^2^)	0.28	0.28 (0.04–0.53)	67 (0, 133)	0.052	168	-399, +532
	RBCF (%)	0.51	0.51 (0.27–0.69)	1.4 (-0.0, 2.8)	0.061	3.6	-8.5, +11.2
	PBR (μm)	0.33	0.33 (0.08–0.56)	-0.10 (-0.17, -0.03)	0.008	0.18	-0.59, +0.39
Inter-rater^a^	VD (μm/mm^2^)	0.33	0.26 (0.03–0.50)	122 (-2, 213)	0.013	180	-456, +578
	RBCF (%)	0.03	0.03 (0.00–0.32)	3.5 (1.0, 5. 9)	0.014	5.3	-11.9, +18.2
	PBR (μm)	0.37	0.26 (0.01–0.51)	-0.15 (-0.25, -0.01)	0.007	0.20	-0.64, +0.47

VD: Vascular Density. RBCF: Red Blood Cell Filling. PBR: Perfused Boundary Region.

^a^ Studies were based on two measurements.

^b^ The mean difference reports the mean difference between second and first measurements of the same rater in the intra-rater study and the second and first raters in the inter-rater study.

### Intra-rater reproducibility

The intra-rater reproducibility of two measurements one minute apart was poor for all three parameters. The ICC was lowest for the VD and the PBR (respectively, 0.28, 95% CI 0.04, 0.53; and 0.33, 95% CI 0.08, 0.56). The point estimate of the ICC of the RBCF was slightly higher (0.51, 95% CI 0.27, 0.69).

For all three parameters, the Bland-Altman plots did not suggest the presence of interaction between the estimated true value and the difference between measurements or the random error. This means that the reproducibility is not affected by extremely low or extremely high values of the PBR, RBCF, and VD, and that all measures are equally poorly reproducible across their range of values (Fig 2 and S2 Fig).

The intra-rater reproducibility for 6 measurements yielded similar results (S1 Appendix).

### Inter-rater reproducibility

The inter-rater reproducibility of two measurements was poor for all three parameters. The ICC for the VD and the PBR was similar to that in the intra-rater setting (respectively, 0.26, 95% CI 0.03, 0.50; and 0.26, 95% CI 0.01, 0.51). The ICC of the RBCF was extremely low (0.03, 95% CI 0.00, 0.32). In all three parameters, a significant difference was seen between the first and the second measurement, independent of the rater, meaning that repeated measurements tended to consistently produce different readings. In particular: the VD and the RBCF tended to be higher in the second measurement than the first measurement (122, 95% CI -2, 213; and 3.5, 95% CI 1, 5.9, respectively), whereas the PBR tended to be lower in the second measurement than the first measurement (-0.15, 95% CI: -0.25, -0.01). The inter-rater reproducibility for 6 measurements showed ICCs similar to the above and no evidence of a consistent sequence effect, suggesting that the readings do not consistently increase or decrease across 6 measurements (S1 Appendix).

For all three parameters, the Bland-Altman plots did not suggest the presence of interaction between the estimated true value and the difference between measurements or the random error (Fig 2 and S1 Appendix), suggesting that the above reliability features are consistent across their range of values.

### Proofs of concept

The results of the proof of concept studies are presented in Table 3. The observed effect sizes suggested that a meal might increase VD, decrease RBCF, and decrease the PBR, whereas smoking two cigarettes might increase VD, decrease RBCF, and increase the PBR. In neither study did the before-after difference for any of the three parameters reach statistical significance.

**Table 3:** Proofs of concept.

Study (sample)	Parameter	Before	After	Estimated difference	P-value	Sample size required for significance
Before vs after a meal (N = 50)	VD (μm/mm2)	724 (182)	763 (170)	39 (-29, 107)	0.257	748
	RBCF (%)	73.0 (4.5)	72.4 (3.8)	-0.6 (-2.2, 1.0)	0.427	1481
	PBR (μm)	1.88 (0.19)	1.84 (0.17)	-0.03 (-0.11, 0.04)	0.350	1101
Before vs after smoking (N = 21)	VD (μm/mm^2^)	704 (198)	730 (234)	26 (-117, 170)	0.707	1640
	RBCF (%)	74.1 (4.8)	71.8 (5.0)	-2.3 (-5.1, 0.5)	0.099	111
	PBR (μm)	1.84 (0.22)	1.88 (0.28)	0.04 (-0.11, 0.20)	0.530	600

VD: Vascular Density. RBCF: Red Blood Cell Filling. PBR: Perfused Boundary Region.

Data are reported as mean (standard deviation). Effect sizes and 95% confidence intervals for before-after difference are derived from a paired t-test.

Atherogenic stimuli such as cigarette smoking are expected to decrease VD and RBCF and to increase the PBR.

Because in these studies two measurements per subject were done by the same rater/researcher, the variance obtained in the intra-rater reproducibility study for 2 measurements was used to calculate the minimum sample size needed to obtain statistical significance for the observed estimated differences. For the study on the effect of a meal, the sample size ranged from 748 to 1481 depending on the microvascular parameter used.

Additional information is included in the S1 Appendix.

## Discussion

Under our testing conditions, the sublingual microcirculation parameters VD, RBCF, and PBR display poor reproducibility, that seems to originate from poor correlation between measurements on the same subject and high residual variance over the whole range of values rather than association between the true value and either variance or the difference between repeated measurements (S1 Fig).

Our analyses were performed on several samples and attempted to replicate conditions common in both clinical practice and clinical research, seeking to provide an insight into their applicability to both settings.

Several considerations can be made about the implications of our findings on the interpretation of measurements based these parameters. First, their measurement error suggests that caution should be exerted when attempting to detect clinically relevant intra-individual differences or classify patients into clinically different groups. In particular, the SEM of the best investigated parameter, the PBR, was 0.18 μm for the intra-rater setting and 0.20 μm for the inter-rater setting. These values are very close to all statistically significant differences in PBR between clinically different groups of patients reported by previous studies: these differences range from 0.2 μm for the difference between patients with premature cardiovascular disease and healthy controls reported by Mulders et al.[9] to the difference of 0.3 μm between dialysis patients and healthy controls reported by Vlahu et al.[24] It therefore seems unlikely that such differences could be detected when measuring the PBR on a single patient in a clinical setting. Second, the parameter readings seem to be somewhat affected by the order of the measurements. Third, using several measurements by different raters does not affect the reproducibility significantly compared to using several measurements by the same rater.

Another important consideration concerns the use of these parameters in research studies. Our results show that very large samples would be needed to achieve statistical significance. Since the probability of finding false correlations with large sample sizes is extremely high even when using parameters with moderate to high reproducibility, particular care should be taken when determining whether any reported correlations involving these parameters reflect true biological differences. This could explain why so much variation has been reported in published studies using these parameters and why their outcomes do not seem to be reproducible.

To the best of our knowledge, this is the first study to systematically evaluate the reproducibility of these parameters. Interestingly, a number of studies have assessed the reproducibility of parameters describing vascular density defined and calculated semi-automatically but still based on SDF imaging, and found better reproducibility. A study by Petersen et al. on the cerebral microcirculation of piglets under experimental conditions has reported ICCs above 0.8 for the intra-rater reproducibility of two parameters describing vascular density [25], which suggests that SDF imaging can provide reproducible parameters under experimental, well-controlled conditions. The need to standardize such conditions, however, confirms that this technology might be more suitable for scientific research than clinical application. Moreover, a study by Hubble et al. assessing the variability across several conditions of a semi-automated method of estimating vascular density from SDF video recordings has concluded that the estimated density can vary for up to over 50% depending on the sublingual location used for measurement, and that this variation increases as vessel size increases [26]. This suggests that reproducibility might be increased by parameters or investigations focusing on smaller vessels, such as proper capillaries (≤10 μm).

A number of limitations apply to our study. First, the conclusions of a reproducibility study can only be applied to populations with the same features as that in which the reproducibility parameters were estimated. In our case, subjects were partly healthy volunteers and partly participants drawn from the HELIUS study. While the HELIUS study population is ethnically diverse, it only includes individuals aged 18 to 70 and is not selected based on specific health profiles [15, 27]. Since parameters with comparable definition have been reported to have prognostic value in critically ill patients [2], it is possible that such parameters only display clinically relevant and reproducible changes in that selected population, but not in a relatively healthy population. Second, in our studies we used Microvision videomicroscopes. Other videomicroscopes have been used in combination with the Glycocheck software to estimate the sublingual microcirculation parameters we studied. If measurements obtained from different videomicroscopes are not be comparable to each other, this might have affected our findings. In particular, image acquisition stabilization might reduce the accuracy of SDF measurement techniques [2], and standardizing the measurement procedure might not be sufficient to control it in a clinical setting; the use of an image acquisition stabilizer might therefore be necessary [28]. Third, we did not control for environmental temperature, temperature at the tip at the camera, or participant body temperature (including fever). A vascular reaction to temperature variations might have increased the moment-to-moment variability of microvascular function, thus reducing reproducibility.

In conclusion, the Sidestream Darkfield-derived sublingual microcirculation parameters seem poorly reproducible in their current form and under the conditions described in the present study. Their reproducibility might be increased by restricting their use to selected populations such as the critically ill, redefining the parameters to only explore microvascular diameters in the anatomic capillary range, or improving the measurement procedure by using an image acquisition stabilizer and verifying the impact of videomicroscope specifics and temperature. When these parameters are used in a research setting, it may be advisable to use a large sample size.

## Supporting information

S1 Appendix Complete results of the reproducibility analysis for the three sublingual microcirculation parameters. VD: Vascular Density. RBCF: Red Blood Cell Filling. PBR: Perfused Boundary Region. SEM: Standard Error of Measurement. Confidence intervals are included in the estimates for the ICC and the sequence effect size. **Calculation of the required sample size. Example: application to the PBR in the proof of concept on the effect of a meal**.
(DOCX)

S2 Appendix Supplementary information regarding the calculation of the SDF-derived microcirculation parameters Vascular Density, Red Blood Cell Filling, and Perfused Boundary Region and the measurement procedure in the HELIUS study. (DOCX)

S3 Appendix Comma-separated values dataset of all data used for the analysis in wide format. (CSV)

S4 Appendix Standard Operating Procedure for the measurement of the sublingual microvascular parameters in the HELIUS study. (PDF)

S1 Fig Possible causes of inadequate reproducibility of a measurement method. (TIF)

S2 Fig Scatter plots and Bland-Altman plots for the intra-rater and inter-rater analyses of the parameters VD and RBCF. (A) Scatter plot of the intra-rater RBCF data. (B) Bland-Altman plot of the inter-rater RBCF data. (C) Scatter plot of the inter-rater RBCF data. (D) Bland-Altman plot of the inter-rater RBCF data. (E) Scatter plot of the intra-rater VD data. (F) Bland-Altman plot of the inter-rater VD data. (G) Scatter plot of the inter-rater VD data. (H) Bland-Altman plot of the inter-rater VD data.
(TIF)
