# Supplementary material for: Reproducibility of sublingual microcirculation parameters obtained from sidestream darkfield imaging
Source: PLoS One. 2019 Mar 14;14(3):e0213175. doi: 10.1371/journal.pone.0213175 (PMC6417651; doi:10.1371/journal.pone.0213175)
Supplement: S1 Appendix — VD: Vascular Density. RBCF: Red Blood Cell Filling. PBR: Perfused Boundary Region. SEM: Standard Error of Measurement. Confidence intervals are included in the estimates for the ICC and the sequence effect size. Calculation of the required sample size. Example: application to the PBR in the proof of concept on the effect of a meal. (DOCX) [file pone.0213175.s001.docx]

**Complete results of the reproducibility analysis for the three sublingual microcirculation parameters.**

| Context | Parameter (unit) | Correlation coefficient^a^ | Inter-subject variance^b^ | Inter-rater variance^b^ | Residual variance^b^ | ICC | Sequence effect^c^ | Sequence effect *p* | SEM | Limits of agreement  (lower, upper)^d^ |
| --- | --- | --- | --- | --- | --- | --- | --- | --- | --- | --- |
| Intra-rater,  2 measurements | VD (µm/mm^2^) | 0.28 | 11036 | - | 28209 | 0.28 (0.04-0.53) | 67 (0, 133) | 0.052 | 168 | -399, +532 |
|  | RBCF (%) | 0.51 | 12.89 | - | 12.61 | 0.51 (0.27-0.69) | 1.4 (-0.0, 2.8) | 0.061 | 3.6 | -8.5, +11.2 |
|  | PBR (µm) | 0.33 | 0.02 | - | 0.03 | 0.33 (0.08-0.56) | -0.10 (-0.17, -0.03) | 0.008 | 0.18 | -0.59, +0.39 |
| Inter-rater,  2 measurements | VD (µm/mm^2^) | 0.33 | 14553 | 8331 | 32563 | 0.26 (0.03-0.50) | 122 (-2, 213) | 0.013 | 180 | -456, +578 |
|  | RBCF (%) | 0.03 | 1.00 | 2.06 | 28.21 | 0.03 (0.00-0.32) | 3.5 (1.0, 5. 9) | 0.014 | 5.3 | -11.9, +18.2 |
|  | PBR (µm) | 0.37 | 0.02 | 0.01 | 0.04 | 0.26 (0.01-0.51) | -0.15 (-0.25, -0.01) | 0.007 | 0.20 | -0.64, +0.47 |
| Intra-rater,  6 measurements | VD (µm/mm^2^) | 0.23 | 8294 | - | 30182 | 0.22 (0.04-0.38) | -16 (-33, 2) | 0.090 | 174 | - |
|  | RBCF (%) | 0.20 | 4.17 | - | 17.23 | 0.20 (0.02-0.37) | -0.2 (-0.6, 0.2) | 0.402 | 4.2 | - |
|  | PBR (µm) | 0.23 | 0.01 | - | 0.04 | 0.24 (0.05-0.42) | 0.00 (-0.02, 0.02) | 0.810 | 0.21 | - |

VD: Vascular Density. RBCF: Red Blood Cell Filling. PBR: Perfused Boundary Region. SEM: Standard Error of Measurement. Confidence intervals are included in the estimates for the ICC and the sequence effect size.

^a^ The correlation coefficient is Pearson’s r. In the analysis using six measurements, the average of the correlation coefficients of all combinations of two measurements was used.

^b^ Variances are expressed in squared units of the parameter considered. The inter-rater variance was only calculated in inter-rater studies.

^c^ The sequence effect is expressed in units of the parameter considered and reports the difference between second and first measurement in the studies comparing 2 measurements, the difference between each additional measurement and the previous one in the study comparing 6 measurement.

^d^ The Bland-Altman limits of agreement cannot be calculated in studies including more than 2 measurements.

**Calculation of the required sample size**

The required sample size to detect the minimal difference between two groups can be calculated using the standard formula [1, 2]:

$$N=2\left(Z_{\alpha}+Z_{\beta}\right)^{2}*\frac{S_{\delta}^{2}}{\delta^{2}}$$

Where $Z_{\alpha}$ = 1.96 for an alpha error of 0.05, $Z_{\beta}$ = 0.84 for a power of 0.80, $\delta$ is the minimal detectable difference in the measurement of interest and $S_{\delta}^{2}$ the sum of the variances due to different measurement components.

In our study, the sum of the variance components is the sum of two components: the variance due to measurement, which will be encountered twice and therefore multiplied by 2, and variance due to true differences between the situation before and after, which is also encountered twice, and therefore multiplied by 2. However, the components before and after are also correlated with each other, and their correlation coefficient is also considered in the calculation. Therefore,

$$S_{\delta}^{2}=\left(2*S_{measurement}^{2}\right)+2\left(S_{residual}^{2}\right)\left(1-r\right)$$

**Example: application to the PBR in the proof of concept on the effect of a meal**

The sum of variances due to different measurement components is obtained by using the variances obtained in the mixed effects model from the intra-rater study, obtained from Table S1 (with precision to the third decimal figure, without rounding):

$$\begin{matrix} S_{measurement}^{2}=0.031 \\ S_{residual}^{2}=0.015 \\ r=0.333 \end{matrix}$$

The minimal detectable difference $\delta$ is considered to be the before-after difference detected in our sample: therefore, -0.0343 mm (Table S1).

Therefore,

$$\begin{matrix} S_{\delta}^{2}=\left(2*S_{measurement}^{2}\right)+2\left(S_{residual}^{2}\right)\left(1-r\right) \\ =\left(2*0.031\right)+2\left(0.015\right)\left(1-0.333\right) \\ =0.0621+0.0305*(0.667) \\ =0.0621+0.0203 \\ =0.0825 \end{matrix}$$

Inserting these values into the original formula along with the z-values, we obtain the required sample size of 1107:

$$\begin{matrix} N=2*\left(1.96+0.84\right)^{2}*\frac{0.0825}{\left(-0.0343\right)^{2}}; \\ =2*7.84*\frac{0.0825}{0.00117}; \\ =15.7*70.5; \\ =1107 \end{matrix}$$

**References for the sample size calculation**

1. de Groot E, Zwinderman AH, van der Steen AF, Ackerstaff RG, Montauban van Swijndregt AD, Bom N, et al. Variance components analysis of carotid and femoral intima-media thickness measurements. REGRESS Study Group, Interuniversity Cardiology Institute of The Netherlands, Utrecht, The Netherlands. Regression Growth Evaluation Statin Study. Ultrasound Med Biol. 1998;24(6):825-32.

2. Neter J., Wasserman W., Kutner MH. Applied linear statistical models. 3rd ed. Boston1990.
