# Supplementary material for: Reproducibility of sublingual microcirculation parameters obtained from sidestream darkfield imaging
Source: PLoS One. 2019 Mar 14;14(3):e0213175. doi: 10.1371/journal.pone.0213175 (PMC6417651; doi:10.1371/journal.pone.0213175)
Supplement: S2 Appendix — (DOCX) [file pone.0213175.s002.docx]

**S2 File. Supplementary information regarding the calculation of the SDF-derived microcirculation parameters Vascular Density, Red Blood Cell Filling, and Perfused Boundary Region and the measurement procedure in the HELIUS study.**

The measurement procedure and the calculation of the PBR have been described in detail elsewhere [1, 2]. In short, the probe of a hand-held sidestream darkfield (SDF) videomicroscope (MicroVision Medical Inc., Wallingford, PA), covered by a disposable cap, is placed on the sublingual mucosa of the subject for approximately 2 minutes to obtain video recordings of the sublingual microvasculature. Subsequently, an analysis software (GlycoCheck ICU, Glycocheck BV, Maastricht, the Netherlands) automatically calculates the sublingual microcirculation parameters within 5 minutes.

Based on the detection of the haemoglobin contained in erythrocytes, the software selects vascular segments spaced 10 μm from each other. It then establishes 21 line markers within each vascular segment, spaced 0.5 μm from each other, and selects at least 3,000 vascular segments in which at least 11 of the 21 markers (52%) detect an erythrocyte in a single frame. This quality check is meant to minimise the impact of hematocrit on the parameters calculated subsequently. The number of 10 μm vascular segments that pass this check is used to calculate the first output parameter, the Vascular Density, defined as the microvascular length found to be perfused according to the above criterion, expressed in micron per square millimeter of sublingual surface explored by the videomicroscope. While some previous studies have used the term Capillary Density for this parameter [3, 4] or similar ones [5] we adopt the term Vascular Density because some of the microvessels in this range have too large a diameter to be anatomically defined as capillaries.

In each of the selected vascular segments, for each of the 21 line markers, radial intensity profiles (width of the flowing erythrocyte column) are obtained in 40 frames, for a total of 840 intensity profiles per vascular segment. At this stage, a second quality check is performed, based on minimum RBC width, position of the column, and signal-to-noise ratio. The line markers that pass this check are used to calculate the second output parameter, the Red Blood Cell Filling (RBCF), defined as the mean percentage of the line markers that detect erythrocytes over the 40 frames of the recording. Because each line marker is placed at the center of a 0.5 μm long longitudinal vascular segment, the RBCF can be interpreted as the proportion of the length of blood vessels that is occupied by erythrocytes over a short time duration.

Lastly, for each vascular segment, the distribution of the measured erythrocyte widths is plotted. For those vascular segments that pass a final check for the linearity of this distribution, e.g. an R-squared of at least 0.7 for the linear regression drawn from the 25^th^ and 75^th^ percentiles, the estimated outer edge of the erythrocyte-perfused lumen is calculated by linear extrapolation of the 50^th^ and the 75^th^ percentile of the erythrocyte column width distribution. Then, the Perfused Boundary Region (PBR) is calculated as the difference (outer edge of the erythrocyte-perfused lumen) – (median erythrocyte column width), which is then divided by two because the PBR is present at both sides of the erythrocyte column. This parameter therefore measures the dynamic lateral erythrocyte movement into the glycocalyx in μm, and estimates the accessibility to erythrocytes of a superficial component of the glycocalyx which is erythrocyte-permeable. The PBR is therefore inversely related to the glycocalyx size: if increased, it reflects a disturbance of the glycocalyx structure or function.

Since the thickness of the glycocalyx is proportional to capillary diameter, recordings are made only on capillaries of diameter (as estimated by median erythrocyte column width) between 5 and 25 μm. Since the distribution of measured capillaries might vary per individual and per measurement location, a single median PBR per 1 μm – capillary diameter class is calculated, and the average of the resulting 26 PBRs is used as PBR for that subject.

Image acquisition is semi-automated, as it requires the investigator to choose the sublingual location for the videomicroscope, and moving to a different location might be needed for the analysis software to collect enough frames for the calculation of the sublingual parameters. The calculation itself is fully automated and blinded to the investigator.

Image acquisition in HELIUS study participants occurred at the same moment in the standard sequence of the physical examination of the HELIUS data collection. Physical examination took place in two locations in the city of Amsterdam using three devices provided by Glycocheck, BV. Each videomicroscope was calibrated daily around 8:00 according to the manufacturer’s instructions. Participants had fasted and discontinued all medications for at least eight hours. They underwent the measurement of sublingual microcirculation parameters in the morning between 8:00 and 12:00 after a 60 minutes history taking and non-invasive physical examination (body circumferences, weight and height, blood pressure using a sphygmomanometer, and electrocardiogram). They were in sitting position on a standard phlebotomy chair.

A Standardized Operating Procedure (SOP) specifically developed for sublingual microcirculation parameters in the HELIUS studies was then applied consistently by all researchers; the SOP included a training program that all researchers had to complete before using the device on study participants. In short, all participants received a description of how the device operated and were requested to relax prior to the measurement. The researcher then removed a new transparent plastic cap, provided by the manufacturer, from its slip case and placed it on the videomicroscope; introduced the videomicroscope under the participant’s tongue in close proximity to the frenulum linguae; started the automatic video recording, and progressively decreased the pressure exerted on the sublingual mucosa to the minimum necessary to keep the recording in focus (as monitored by the software), so as to allow erythrocytes to flow freely and minimize the patient’s discomfort. In each participant, the minimum number of sublingual locations necessary for the software to collect sufficient frames for PBR calculation was used; e.g., a single sublingual location was used unless the software indicated more frames were needed for PBR calculation, in which case the videomicroscope was moved to a second location until recording resumed, and kept still until the software closed the recording by signaling that the required number of frames had been reached. Using this procedure, recording time ranged between 1.30 and 10 minutes. At the end of the recording, the plastic cap was discarded.

**References**

1. Lee DH, Dane MJ, van den Berg BM, Boels MG, van Teeffelen JW, de Mutsert R, et al. Deeper penetration of erythrocytes into the endothelial glycocalyx is associated with impaired microvascular perfusion. PLoS One. 2014;9(5):e96477. doi: 10.1371/journal.pone.0096477.

2. Dane MJ, Khairoun M, Lee DH, van den Berg BM, Eskens BJ, Boels MG, et al. Association of kidney function with changes in the endothelial surface layer. Clin J Am Soc Nephrol. 2014;9(4):698-704. doi: 10.2215/cjn.08160813.

3. Groen BB, Hamer HM, Snijders T, van Kranenburg J, Frijns D, Vink H, et al. Skeletal muscle capillary density and microvascular function are compromised with aging and type 2 diabetes. J Appl Physiol (1985). 2014;116(8):998-1005. doi: 10.1152/japplphysiol.00919.2013.

4. Gu YM, Wang S, Zhang L, Liu YP, Thijs L, Petit T, et al. Characteristics and Determinants of the Sublingual Microcirculation in Populations of Different Ethnicity. Hypertension. 2015. doi: 10.1161/hypertensionaha.114.05119.

5. Hubble SM, Kyte HL, Gooding K, Shore AC. Variability in sublingual microvessel density and flow measurements in healthy volunteers. Microcirculation. 2009;16(2):183-91. doi: 10.1080/10739680802461935.
