# Supplementary material for: Reproducibility of sublingual microcirculation parameters obtained from sidestream darkfield imaging
Source: PLoS One. 2019 Mar 14;14(3):e0213175. doi: 10.1371/journal.pone.0213175 (PMC6417651; doi:10.1371/journal.pone.0213175)
Supplement: S4 Appendix — (PDF) [file pone.0213175.s004.pdf]

Supplementary File S4

Excerpt of the internal measurement protocol (Standard Operating Procedure) for the sublingual microvascular parameters in the HELIUS study, 2012-2014 (English translation, followed by the original Dutch)

Screenshots of copyrighted software have been obscured.

SOP HELIUS GlycoCheck

The glycocalyx is a layer of molecules that lines and protects the interior surface of the blood vessels. This layer has multiple biochemical functions. The thickness of the glycocalyx could play a role in the development of cardiovascular diseases such as myocardial infarction and stroke.
The GlycoCheck system measures the thickness of the glycocalyx: a vidrecords a video of the microvasculature under the tongue and in the lingual frenulum and of the blood flow passing through. Then the software uses the recordings to automatically estimate the thickness of the glycocalyx. The thickness of the glycocalyx of the sublingual microvessels reflects the thickness of the glycocalyx of all blood vessels in the body. In the HELIUS study, we examined the possibility of a link between glycocalyx thickness and other cardiovascular parameters such as blood pressure, ethnicity, and incidence of cardiovascular diseases, such as myocardial infarction, and diabetes later in life.

2 Measurement of the participant

2a. Introduction

Upon arrival of the participant:
Invite the participant to take a seat, the researcher sits at the computer. All you need are four short sentences to introduce the test (the most important are underlined):

a. ***In this room, the first test includes a new device: a camera that records a video of the blood vessels under your tongue;***
b. ***This way we can <u>study the small superficial blood vessels</u> under your tongue*** (you may also point at your wrist to show that superficial blood vessels are visible).
    - If the participant has any further questions, you may add that by studying these blood vessels we gather information on all the other blood vessels in the body that are similar to the blood vessels under the tongue. You may also add that this information can be used to study diseases such as high blood pressure, cardiovascular diseases and diabetes.
c. ***The test will only take <u>a few minutes</u> and is fully <u>automated. The test is painless</u>.***
d. ***This is a new type of measurement which is still in the experimental stage, therefore, you will not receive the results.***

2b. Registration of the participant

- Upon arrival of the participant, he/she should be registered in the programme.
- For each participant, take the following steps in the second column (*Patient registration*) (**B**):
  - Under *Auto search modus*, choose *Add new patient*.
  - Fill in the following details:
    - In the field *OriginalPatient ID*, fill in the participant's HELIUS number.
    - In the field *Date of Birth*, fill in the participant's date of birth in the format "dd-mm-yyyy" (as indicated next to the field).
    - In the fields *Surname* and *First Name* you fill in the initials (first letters of the surname and the first name) (no more than 3 letters per field).
    - Under *Gender* you select the gender of the participant.
  - Click on *Add patient* and then click on *Admission* (left to *Add patient*).

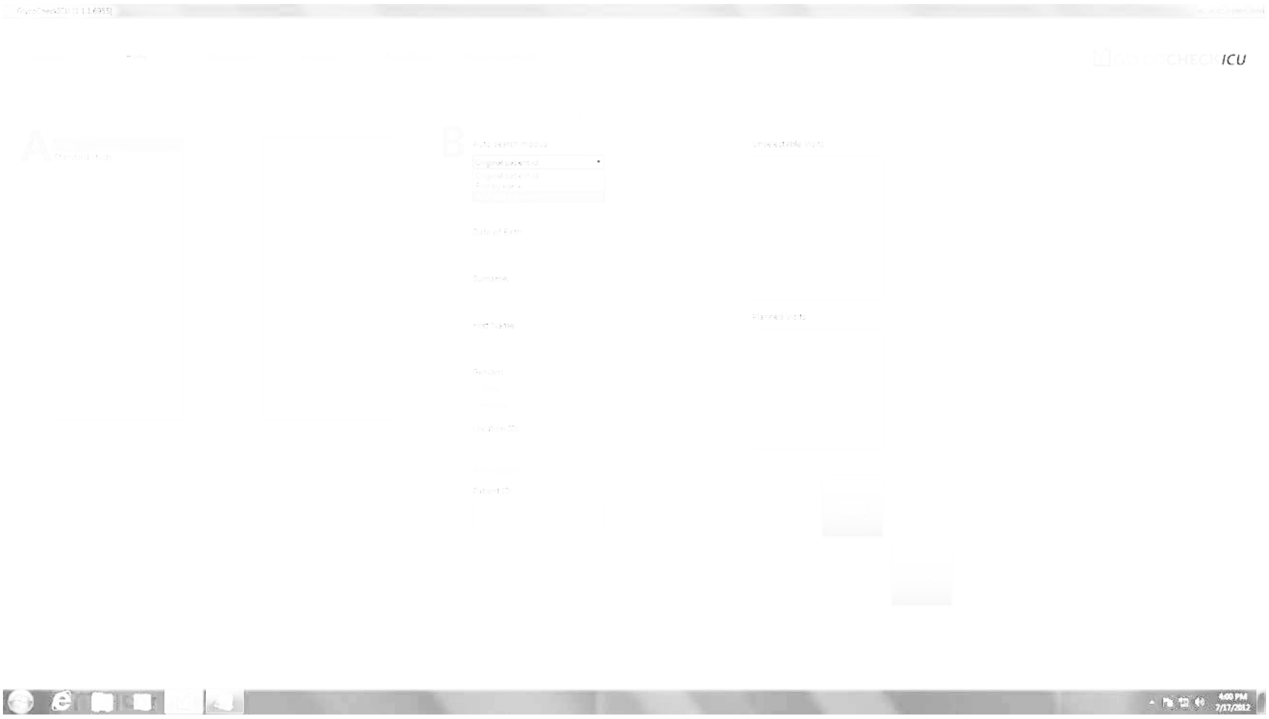

- When the programme indicates that there is already a patient with that number in the list, fill in the participant's HELIUS number, followed by an "x", for example 123456x instead of 123456) and record this change on the day sheet.
- The name of the newly added participant will appear in the list of column 2, *Active patients* (if the list includes many participants, the name of the participant will not be immediately visible, but you can perform an automatic search) → go to section **2c. Start of the test**.

2c. Start of the test

1) Take the package of a camera cap (**A**) from the right side of the desk drawer, where the new caps are stored. Show the package to the participant and explain that you are ***now placing a new clean cap on the camera and that you do this for every participant***.
   Remove the old cap, which is being used as protection, from the camera and place it on the table. Open the paper package without touching the cap by removing the plastic layer without tearing the paper. Place the paper package in the tray under the desk. The old paper packages will be re-used.
   Firmly place the cap on the camera; you will hear a click and the light at the back of the camera should turn green (green arrow in the figure on the right). If the light does not turn green immediately, try attaching the cap with more force.

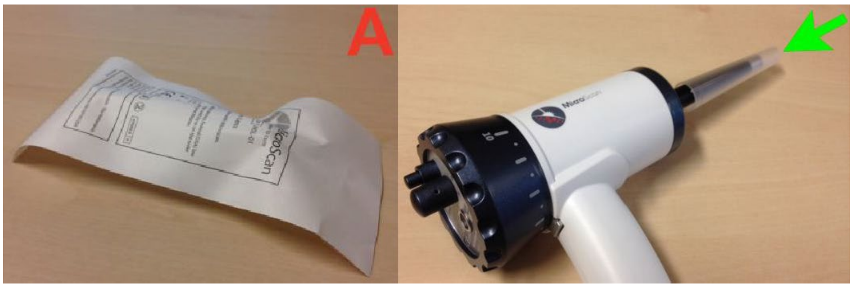

2) Ask the participant to:
   a. ***swallow as much of the saliva in their mouth as possible so the mouth will be drier (this will make it easier to record).*** If the participant says that their mouth is already dry, explain that there is always saliva present in the mouth, even when we think that this is not the case, and ask them to try it anyway.
   b. ***position their head and back firmly against the back of the chair.***
      i. The chair and the head support should be adjusted to the participant to ensure that the participant's neck is not tilted backwards, but straight and aligned with the torso and the head. If this is not the case, the tongue and the saliva will move to the back of the throat and the participant will cough/swallow often during the recording.
   c. ***open the mouth wide and raise the tongue for just one second*** (after which they will be able to relax the tongue).
3) Find a stable but comfortable position for yourself (for example, placing the weight of the body fully on one of the two legs).
   Gently place the camera in the participant's mouth approaching from the side.
   Then let the camera rest on the bottom teeth of the participant (this will not cause the participant any discomfort).
   Place the tip of the camera on the mucous membrane at the underside of the tongue or in the corner between the lingual frenulum and the underside of the tongue or between the lingual frenulum and the floor of the mouth.
   The camera should be in contact with the mucous membrane throughout. During the entire test, apply moderate pressure without hurting the participant.
4) Ask the participant to ***relax the tongue again***; to be more specific you could use the words "relax the tongue", "soften the tongue" and "rest the tongue on the camera".

5) Next, click on *Perform selected visit* in the fourth column on the screen (**X**).

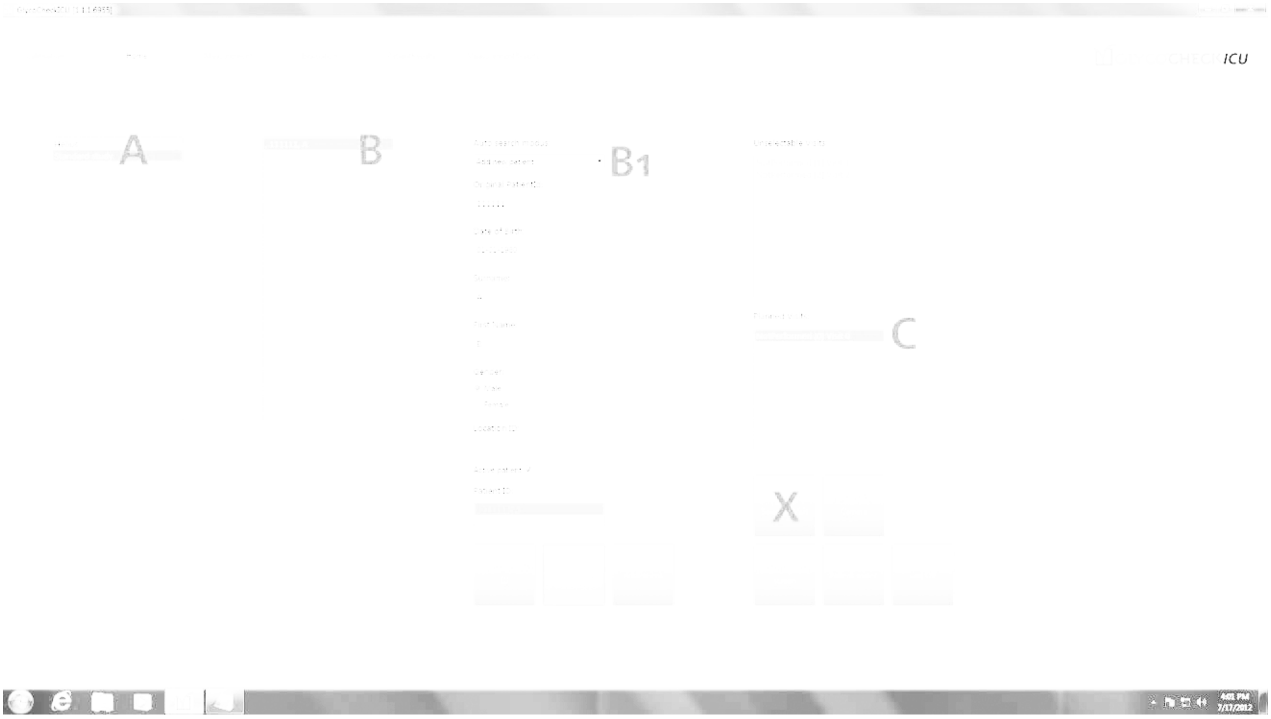

If the message **Encountered a problem** appears on the screen, please check:

- both ends of the cable between the camera and the unit and of the cable between the unit and the computer; both cables should be properly connected at all ends.
- that the GlycoCheck unit is on (switch is in the "on" position and green light).

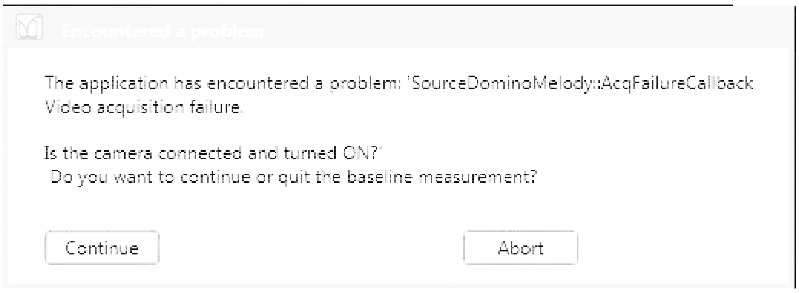

- If you choose "Abort" you will be asked to type some text; a small word, for example "no" or "ok", will suffice.

6) Ask the participant to find a comfortable position for their tongue, so it can relax and rest naturally in the mouth and **tell them they can close their eyes**. In general, the bottom teeth provide sufficient stability, but you could ask the participant to **bite the camera with their teeth** to hold it in a correct position if necessary.

Note

You can use a different measuring method. Ask the participant to:

- sit on a stool or chair in front of the screen;
- place both elbows on the table;
- hold the camera in both hands and place it under their tongue;

- look at the screen and keep the image as steady as possible.

You yourself can adjust the *Focus* ring and the *LIGHT* button and move the camera.

This method could be useful if you find it difficult to keep your leg/arm/hand stable: because the participant is looking at the screen, he/she can use their tongue/hand coordination to keep the image steady.

2d. Measuring

- The screen will look like this:

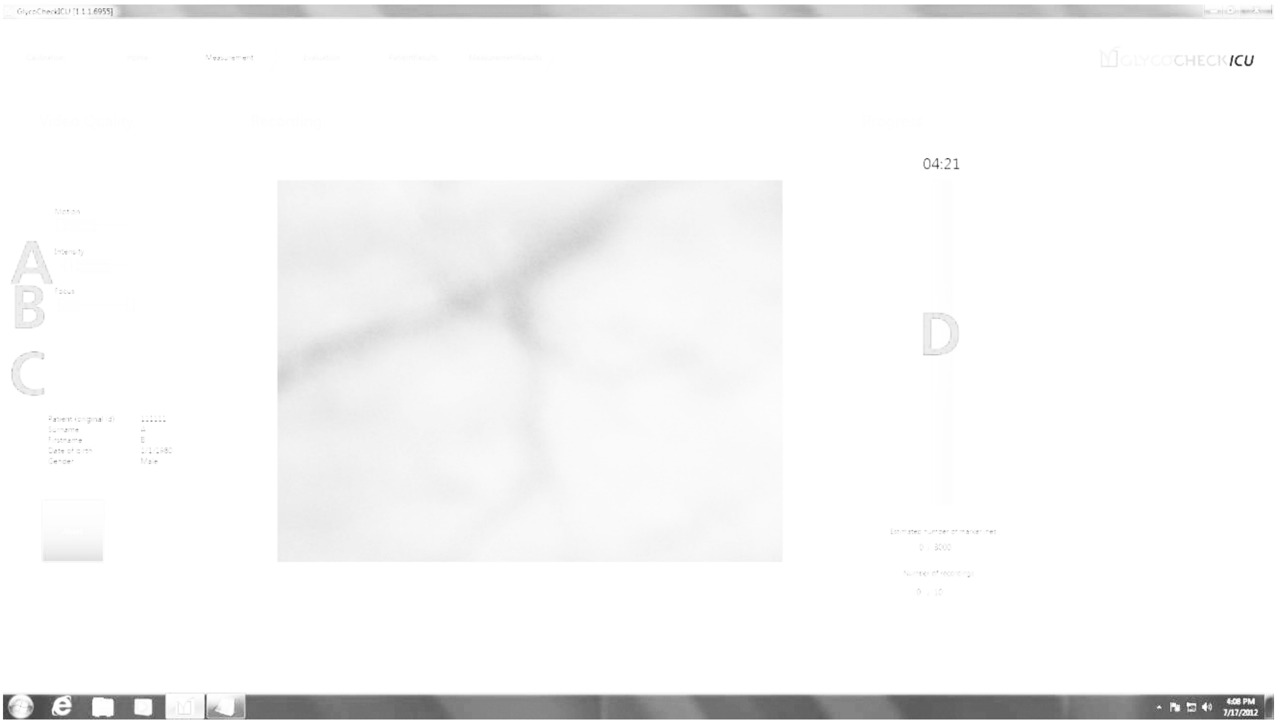

- If you see any stains or water drops on the screen at the start of the test, the water drops could be from the participant's breath and can be either at the inside or at the outside of the cap.
    - Position the camera firmly on the mucous membrane: if the water drops are located on the outside, they will disappear.
    - If you still see the stains and they do not move when you move the camera, the cap is dirty on the inside. Replace the cap.

- Adjust *Intensity* and *Focus* by turning the relevant controls at the back of the camera, similar as during calibration. The image on the screen should become clear, the blood vessels should be visible and the indicators on the screen (**A** and **B**) should be in the green zone. **Intensity is more important than focus:** first adjust the intensity every time it is outside the green zone before you adjust the focus. There is no point adjusting the focus if the intensity is not in the green zone.

Important tips for a good focus.

- If the participant has much saliva, please ask them again to swallow it or give the participant a sip of water.
- Check upon placing the cap that the light turns green **and is green during the entire test**. This is something that can be done with one brief look.
- If after several attempts the cap is not attached properly or if the tip becomes wet with water bubbles that will not go away, just use a new cap.

- Ensure the participant's stability, both physically (the head, but also the rest of the body) and psychologically (the participant should understand that the tongue can be soft and can be lowered and that thereby the recording will take less time: do not start the test when the participant has not grasped this concept yet):
    - let the participant know when he/she is not yet relaxed enough;
    - let the participant know when he/she sits stable and still enough ("it is going well"; "this is perfect", "if you can stay like this, we'll be finished in no time"), so the participant understands what is expected.
- Ensure stability of your arm/wrist (you can apply more pressure to the lower teeth than you might expect.
- The camera should be in contact with the mucous membrane using moderate but firm pressure – in this case you can also apply more pressure than you might expect, without hurting the participant.

- Locate a site that has a sufficient amount of blood vessels.
- Locate a site where the red blood cells appear as **black** and well-defined against a white background instead of grey set against a grey background. Even when you cannot find a site with many large and black vessels, you may get good focus at sites with small, short and curved blood vessels.
- The Focus ring should only be rotated rarely and must be turned rapidly (and only one direction will brighten the image). If you are at a site where the intensity is in a good position, but there is no focus, even after a few fast turns of the Focus ring, it might be better to find a more suitable site than to keep turning at the Focus ring.
- If the screen shows any **air bubbles**, which is often but not always indicated by a red alert in position **C**, or if there are no suitable blood vessels visible, move the camera by either tilting it, moving it or placing it at the other side of the lingual frenulum.
- Sometimes an image in focus can suddenly lose focus. This can occur due to saliva or because the camera has moved a millimetre. Try adjusting the focus as quickly as possible and place the camera deeper in the mouth, or try measuring at another site.

- When both indicators are in the green zone, the software will automatically start recording data and the bar **D** at the right side of the screen starts loading to indicate that the test is running. If the indicators are outside the green zone, the test will stop; try to get back in the green zone and stay as still as possible, the test will automatically proceed.

- This is the ideal situation, with a clear image (the blood cells can be clearly distinguished and they are almost black), all the indicators in the green zone and the bar at the right loading:

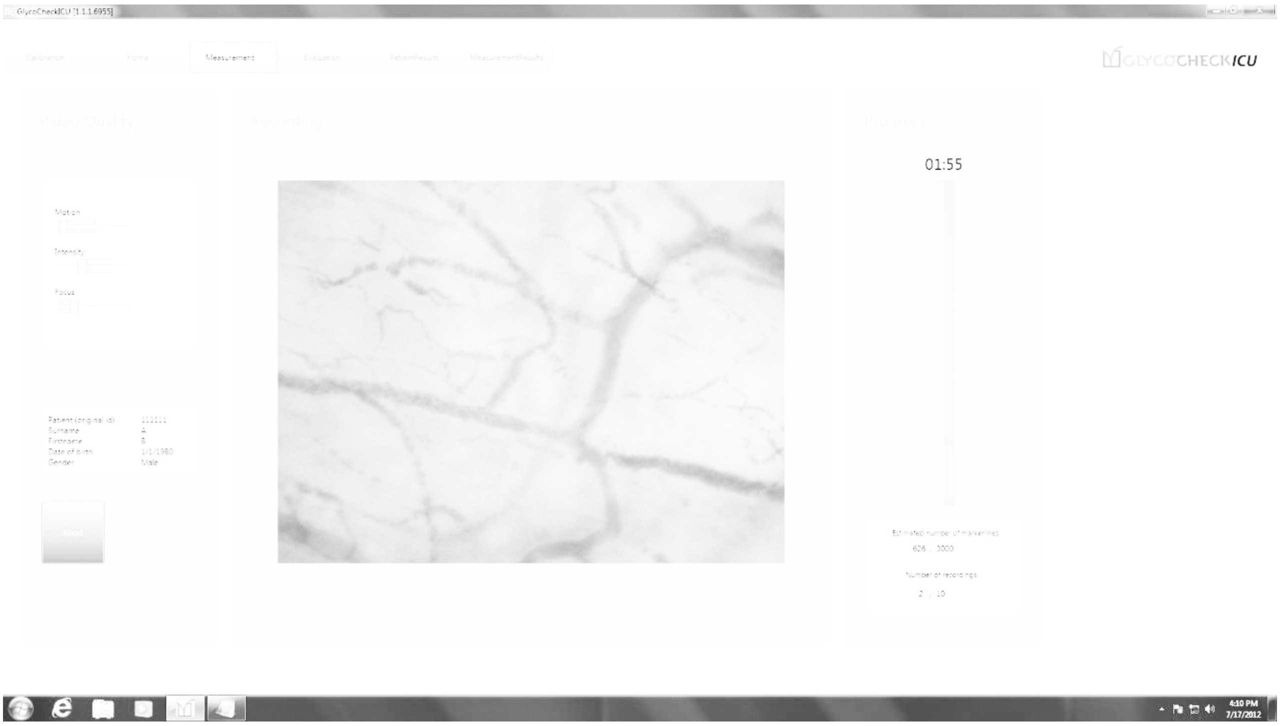

- When the test is finished, the screen *Measurement Evaluation* will appear. There are 3 steps that should be taken immediately.

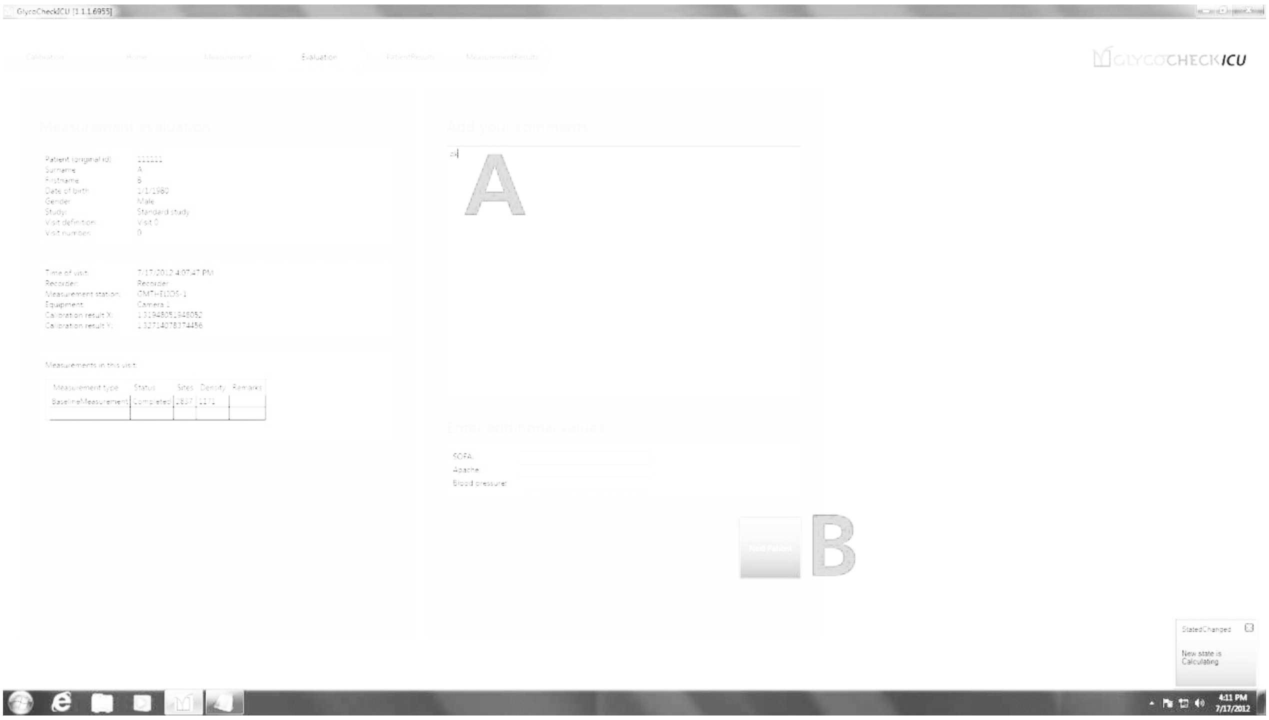

1. Remove the camera from the participant's mouth and discard the cap of the camera in the bin by pressing the *Eject* button at the back of the camera.
2. Click on the white field *Add your comment* (**A**) as soon as possible and type "y" or "ok".
3. Click on *Next Visit* (**B**) and return to the *Home* screen.

- If there is no successful recording after 5 minutes the software will give the options *Continue* and *Stop*. Choose *Stop*. You will be asked to type some text, this time twice. You can type “n” both times and return to the *Home* screen. Do not try again and tell the participant that the test is finished.

- On the day sheet, next to the HELIUS identification code, record a “Y” for the participants who had a successful test, and an “N” for the participants who did not have a successful test.
- In both cases, you can select or add the next participant for the next test on the *Home* screen.

23. End of the test

- Do not discard the used cap. Place it on the plastic mat in the desk drawer. Used caps will be disinfected by Luca at the end of the week and stored in one of the paper packages.

SOP HELIUS GlycoCheck

De glycocalyx is een laag moleculen die de binnenwand van de bloedvaten bedekt en beschermt, met veel biochemische functies. De dikte van de glycocalyx kan een rol spelen bij het ontstaan van cardiovasculaire ziektes zoals hartinfarct en beroertes.
Het GlycoCheck-systeem meet de dikte van de glycocalyx: een camera neemt een video op van de kleine, oppervlakkige bloedvaten onder de tong en in de tongriem en van de bloedstroom erin, terwijl een software de opnames gebruikt om automatisch de dikte van de glycocalyx in te schatten. De dikte van de glycocalyx in de tongbloedvaten weerspiegelt de dikte van de glycocalyx in al de lichamelijke bloedvaten. In HELIUS bestuderen we of er een relatie is tussen de dikte van de glycocalyx en etnische afkomst, de andere cardiovasculaire parameters zoals bloeddruk, en het voorkomen van hart- en vaatziekten zoals hartinfarct en diabetes later in het leven.

2. Deelnemeronderzoek

2a. Introductie

Bij binnenkomst van de deelnemer:
De deelnemer kan gaan zitten en de onderzoeker is bij de computer. Vier korte zinnen zijn genoeg om het onderzoek te introduceren (de belangrijkste worden zijn onderstreept):

a. ***Het eerste onderzoek van deze kamer is een nieuw apparaat: een <u>camera</u> die een <u>video/filmpje</u> gaat opnemen <u>onder uw tong</u>;***
b. ***Het is om de kleine <u>bloedvaten</u> te <u>bestuderen</u> die onder de tong zitten*** (je kunt ook naar je pols wijzen om te laten zien dat oppervlakkige bloedvaten zichtbaar zijn);
   - Indien de deelnemer nog vragen stelt, dan kun je toevoegen dat wij door die tongbloedvaten te bestuderen informatie krijgen over al de andere bloedvaten van het lichaam, die op de tongbloedvaten lijken; en dat deze informatie nuttig kan zijn om ziektes zoals hoge bloeddruk, hart- en vaatziektes en diabetes te onderzoeken.
c. ***Het duurt <u>maar een paar minuten</u> en het is volledig <u>automatisch</u>, u voelt niets.***
d. ***Het gaat om een nieuw soort meting die in de experimentele fase is, en u ontvangt hier geen uitslag van.***

2b. Registratie van de deelnemer

- Zodra de deelnemer in de kamer is, moet hij/zij in het programma worden geregistreerd.
- In de tweede kolom (*Patient registration*) (**B**), doe het volgende voor elke deelnemer:
  - Onder *Auto search modus*, kies *Add new patient.*
  - Vul de volgende gegevens in:
    - Vul het HELIUSnummer van de deelnemer in in het veld *Original PatientID*.
    - Vul de geboortedatum van de deelnemer in in het formaat "dd-mm-jjjj" (zoals naast het veld aangegeven), in het veld *Date of birth*.
    - Vul de initialen (eerste letters van de achter- en voornaam) in in de velden *Surname* en *First Name* (maximaal 3 letters per veld).
    - Selecteer onder *Gender* het geslacht van de deelnemer.
  - Klik op *Add patient* en vervolgens klik op *Admission* (links van *Add patient*).

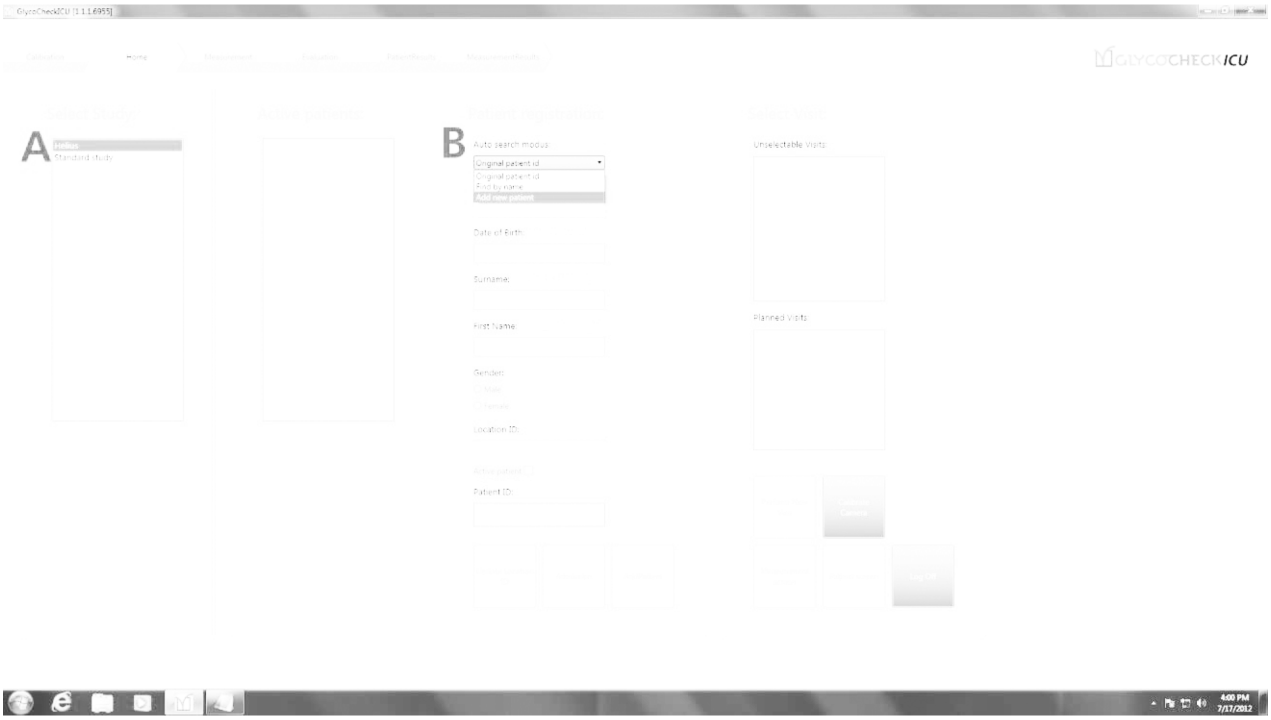

- Als het programma zegt dat een deelnemer met hetzelfde nummer al in de lijst aanwezig is, vul dan het HELIUSnummer van de deelnemer in, gevolgd door een "x" (bijvoorbeeld: 123456x in plaats van 123456) en meld deze verandering op de daglijst.
- De toegevoegde deelnemer verschijnt in de lijst van de kolom 2, *Active patients* (de naam van de deelnemer zal niet onmiddellijk zichtbaar zijn als er al veel patiënten op de lijst aanwezig zijn, maar hij/zij kan automatisch worden gezocht) → ga naar sectie **2c.Onderzoek**.

2c. Begin van het onderzoek

1) Neem het pakje van een camerakapje (**A**) uit de rechterkant van de lade van het bureau, waar schone kapjes klaar staan. Toon het aan de deelnemer, en vertel dat je ***nu een nieuw, schoon kapje op de camera zet, wat bij elke deelnemer gebeurt.***
   Haal het oude kapje dat ter bescherming wordt gebruikt van de camera en leg het op de tafel. Open het papieren pakje zonder het kapje aan te raken door het plasticlaagje te verwijderen, zonder het papier te scheuren. Leg het oude papieren pakje in het bakje onder het bureau. Oude papieren pakje zullen hergebruikt worden.
   Sluit het kapje aan op de camera met een krachtige beweging: je hoort een klik en het licht achter de camera moet groen worden (groene pijl in het figuur rechts). Indien het licht niet meteen groen wordt, probeer het kapje dan met meer kracht aan te sluiten.

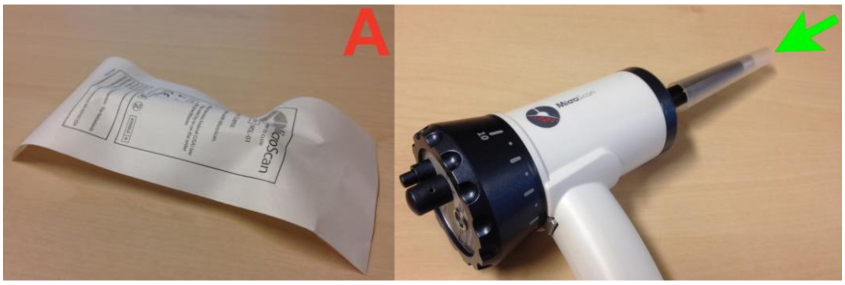

2) Vraag de deelnemer om
   a. ***het speeksel in hun mond zo goed mogelijk door te slikken zodat de mond droger is (wat de opname makkelijker maakt).*** Indien de deelnemer beweert dat de mond al droog is, leg uit dat er altijd speeksel onder in de mond zit, zelfs wanneer we denken dat dat niet zo is, en vraag om het toch te proberen.
   b. ***goed met hoofd en rug tegen de rugleuning te liggen.***
      i. De stoel en de hoofdsteun moeten worden aangepast zodat de keel van de deelnemer niet naar achteren gebogen is, maar parallel is aan torso en hoofd. Anders gaan de tong en het speeksel achter in de keel zitten en de deelnemer zal vaak moeten hoesten/doorslikken tijdens de opname.
   c. ***De mond goed open te doen; en de tong maar één seconde omhoog te doen*** (daarna kunnen ze hem weer ontspannen).

3) Vind een stabiele maar ontspannen positie voor jezelf (bvb het gewicht van het lichaam kan volledig op één van de twee benen rusten).
   Breng voorzichtig de camera in de mond van de deelnemer vanaf de zijkant.
   Laat vervolgens de camera op de ondertanden van de deelnemer rusten (het is niet oncomfortabel voor de deelnemer).
   Zet de punt van de camera tegen het slijmvlies aan de onderkant van de tong of in de hoek tussen de tongriem en de onderkant van de tong of de tongriem en de bodem van de mond.
   De camera moet in contact blijven met het slijmvlies. Oefen tijdens de gehele meting matige druk uit zonder dat dit pijn veroorzaakt bij de deelnemer.

4) Vraag nu de deelnemer om ***de tong weer te ontspannen;*** om duidelijker te zijn kun je de woorden "tong ontspannen", "tong zacht maken" en "tong op de camera laten rusten" gebruiken.

5) Klik vervolgens op *Perform selected visit* in de vierde kolom op het scherm (**X**).

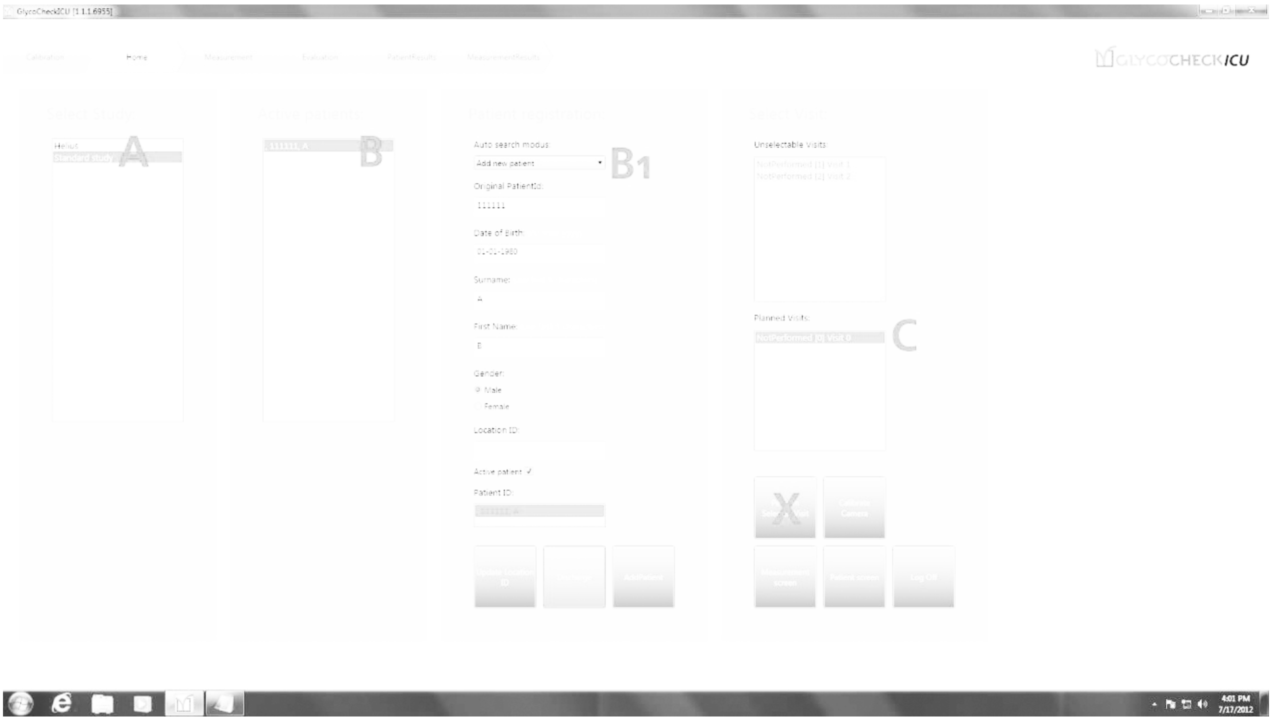

Als er het bericht **Encountered a problem** verschijnt, controleer

- de kabel tussen camera en unit aan beide uiteinden, en de kabel tussen unit en computer aan beide uiteinden; beide kabels moeten op alle punten goed aangesloten zijn.
- dat de glycochek-unit aan is (knopje in "aan"-positie en groen licht).

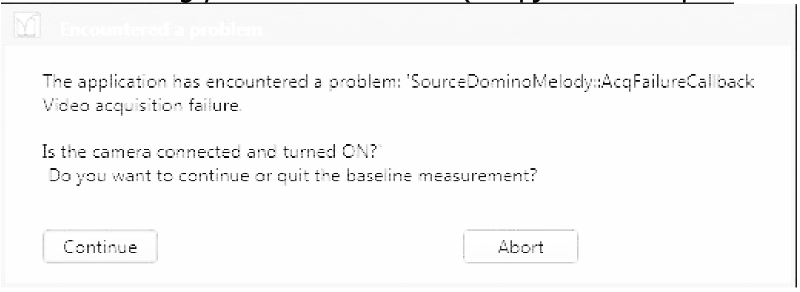

- Als je "abort" kiest, dan word je gevraagd om tekst te typen; een klein woord zoals "no" of "ok" is genoeg.

6) Vraag de deelnemer om een comfortabele positie voor hun tong te vinden, zodat deze rustig en ontspannen kan blijven, en ***zeg dat de ze de ogen dicht kunnen doen.*** Gewoonlijk bieden de ondertanden voldoende stabiliteit, maar indien nodig kun je de deelnemer vragen om op de camera te ***bijten met hun tanden*** om een goede positie te houden.

Aanmerking

Het is ook mogelijk om de meting met een andere methode te verrichten.
Je kunt de deelnemer vragen om

- Op een kruk op stoel vóór het scherm te zitten;
- Beide ellebogen op de tafel te zetten;
- De camera zelf met beide handen vast te houden en onder hun tong te zetten;

- Naar het scherm kijken en het beeld zo stil mogelijk proberen te houden.

Je kunt zelf het Focus-wiel en het *LIGHT*-knopje aanpassen en de camera verplaatsen.
Deze methode is nuttig als je het moeilijk vindt om zelf stabiel met je been/arm/hand te blijven: omdat de deelnemer het scherm ziet, kan hij/zij de coordinatie tussen zijn eigen tong en handen gebruiken om stil te blijven.

2d. Meting

- Het scherm zal er zo uitzien:

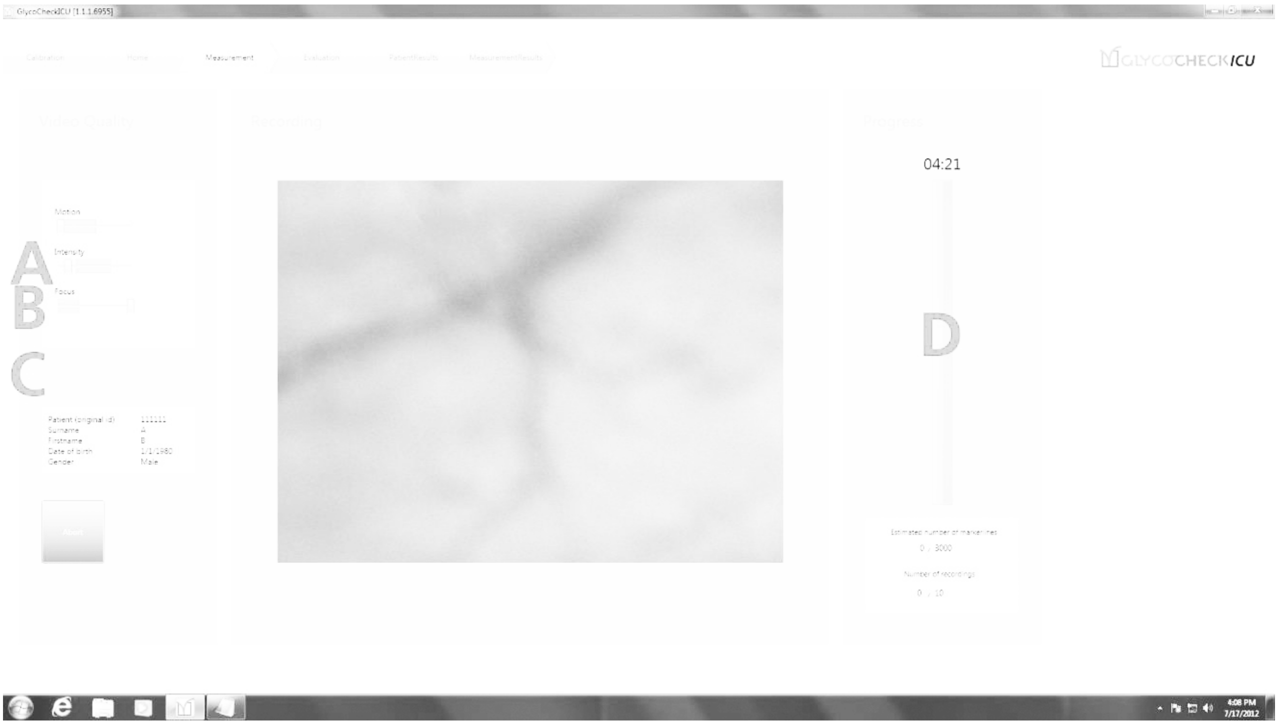

- Als er aan het begin van de meting vlekken of waterdruppels zichtbaar zijn, dan kunnen die waterdruppels uit de ademhaling van de deelnemer komen en ze kunnen of aan de buitenkant van het kapje zijn of aan de binnenkant van het kapje.
    - breng de camera goed in contact met het slijmvlies: als de waterdruppeltjes aan de buitenkant zijn, dan zullen ze weggaan.
    - als de vlekken blijven, en ze niet bewegen wanneer de camera wordt bewogen, dan is het kapje vies aan de binnenkant. Vervang het kapje.

- Pas de Intensity en de Focus aan door de betreffende knoppen achter de camera te draaien, net zoals bij de calibratie. Het beeld moet helder worden met zichtbare bloedvaten, en de indicatoren op het scherm (**A** en **B**) moeten in de groene zone vallen. **De intensiteit heeft voorrang op de focus**: elke keer dat de intensiteit buiten de groene zone valt, pas het aan voordat je de focus aanpast. Het is niet zinvol om de focus aan te passen als de intensiteit niet in de groene zone is.

Belangrijke tips voor een goede focus.

- Als de deelnemer nog heel veel speeksel heeft, vraag opnieuw om het door te slikken of geef de deelnemer een slokje water;
- Kijk bij het bevestigen van het kapje na dat het licht groen is **en groen blijft tijdens het hele onderzoek**. Dat is ook iets wat je heel snel met een kijkje kunt controleren.
- Als een kapje zelfs na heel veel pogingen nog niet goed vastzit, of als de punt nat wordt met waterbelletjes die niet weggaan, pak gewoon een nieuw kapje.

- Zorg voor de stabiliteit van deelnemer, zowel fysiek (het hoofd, maar ook de rest van het lichaam) als psychologisch (de deelnemer moet begrijpen dat de tong zacht kan zijn en zakken, en dat dat de opname ook sneller maakt: niet ineens beginnen met de meting als de deelnemer dit nog nog niet heeft begrepen);
    - laat de deelnemer weten wanneer ze nog niet ontspannen genoeg is.
    - laat de deelnemer weten wanneer ze stabiel en stil genoeg zijn ("nú gaat het goed" , "zó is het bijvoorbeeld perfect", "als u zó blijft zijn we snel klaar"), zodat ze begrijpen wat de bedoeling is.
- Zorg voor de stabiliteit van je arm/pols (je kunt meer druk uitoefenen op de ondertanden van de deelnemer dan je in eerste instantie zou denken);
- De camera moet in contact zijn met het slijmvlies met een matige maar stevige druk – ook in dit geval kun je meer drukt uitoefenen dan je zou denken, zonder pijn te doen.

- Een plek zoeken met genoeg bloedvaten;
- Een plek zoeken waar de bloedlichaampjes er **zwart** en goed onderscheiden uitzien tegen een **wit** achtergrond en niet grijs tegen een grijze achtergrond. Zelfs als je geen plek vindt met veel, grote en zwarte bloedvaten, kun je vaak een goede focus hebben bij plekken met *kleine, korte en kromme bloedvaatjes*.
- Het Focus-wiel dient maar zelden en heel snel te worden gedraaid (en alleen maar een richting maakt het beeld helderer). Als je op een plek bent waar je een goede positie van *Intensity* hebt maar geen focus, ook niet na een paar snelle draaibewegingen van het Focus-wiel, is het zinvoller om elders te gaan zoeken dan om daar langer blijven en lang over het Focus-wiel te doen.
- Als er ***luchtbellen*** op het scherm zijn, wat vaak maar niet altijd door een rood alert wordt aangegeven in de positie **C**, of als er geen goede bloedvaten zichtbaar zijn, verplaats de camera dan door hem óf te kantelen, óf hem te verplaatsen, óf hem aan de andere kant van de tongriem te leggen.
- Soms kan een beeld met focus plotseling geen focus meer hebben. Dat kan komen ofwel door het stromen van speeksel of omdat de camera een millimeter achteruit is gegaan. Probeer de focus snel aan te passen en de camera wat dieper te zetten, anders kun je gewoon ergens anders proberen te meten.

- Zodra beide indicatoren in de groene zone zijn, begint de software automatisch gegevens op te nemen, en de bar **D** aan de rechterkant van het scherm loopt vol om aan te geven dat de meting onderweg is. Indien de indicatoren buiten de groene zone vallen, dan stopt de meting; probeer deze weer in de groene zone te brengen en zo stil mogelijk te blijven, de meting zal dan automatisch verder gaan.

- Dit is dus de ideale situatie, met een helder beeld (de afzonderlijke bloedlichaampjes zijn goed te onderscheiden en ze zijn bijna zwart), al de indicatoren in de groene zone, en de balk rechts die aan het vollopen is:

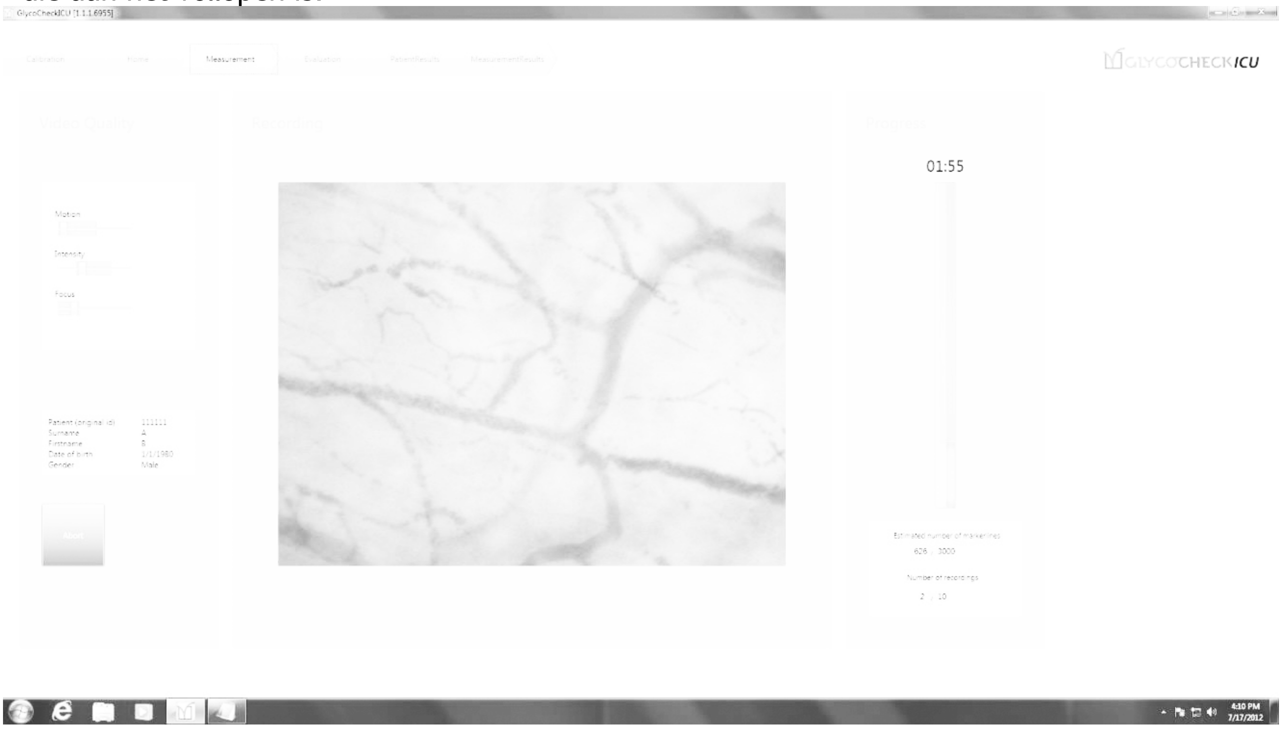

- Zodra de meting klaar is, verschijnt het scherm Measurement Evaluation, waarbij drie dingen **onmiddellijk** dienen te worden gedaan:

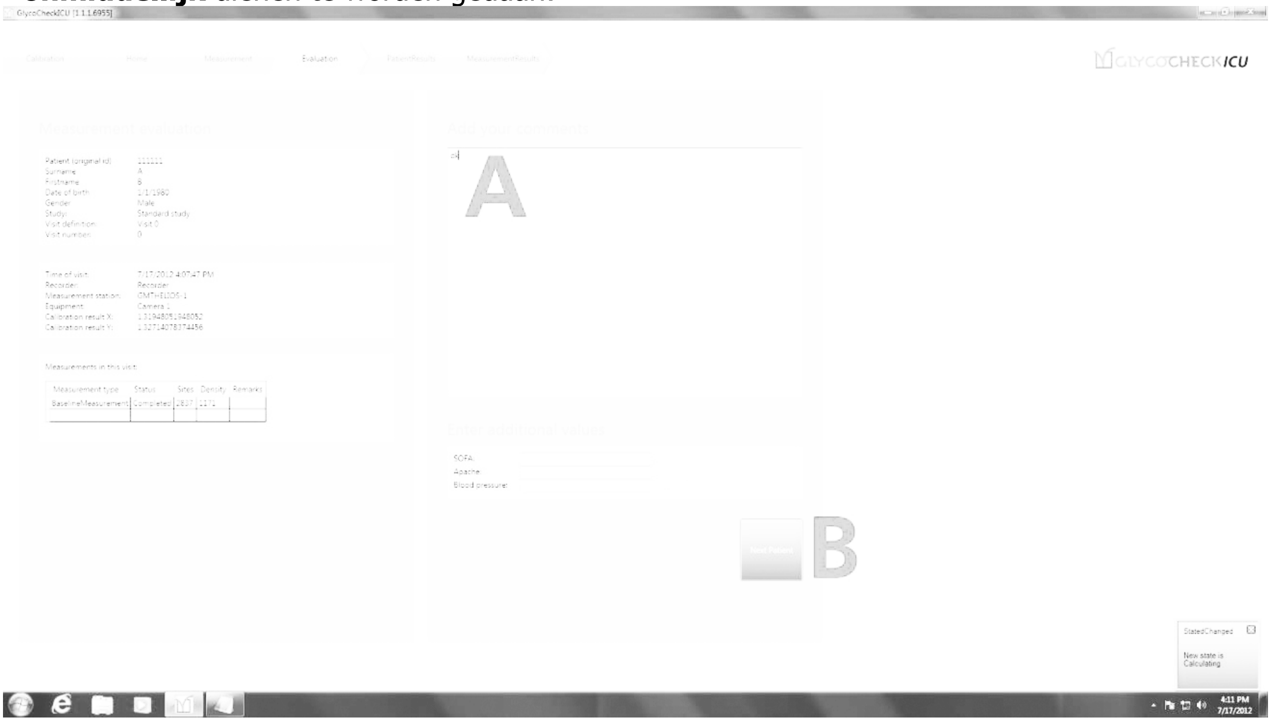

1. Haal de camera uit de mond van de deelnemer en gooi het kapje uit de camera direct in de prullenbak door het knopje *Eject* achter de camera te drukken.
2. Klik *zo snel mogelijk* op het witte veld *Add your comment* (**A**) en typ `j´ of `ok´.
3. Klik op *Next visit* (**B**), en je gaat naar het *Home* screen.

- Indien na 5 minuten nog geen opname is gelukt, zal er een keuze worden aangeboden tussen *Continue* en *Stop*; kies *Stop;* je wordt gevraagd om tekst te typen, maar dan twee keer; beide keren kun je gewoon "n" typen, en je gaat naar het *Home* screen. Probeer het niet opnieuw en vertel de deelnemer dat je klaar bent met dit onderzoek.

- Noteer op de daglijst bij welke deelnemers de meting is gelukt, met een "Y" naast de HELIUScode, en bij welke deelnemers de meting is niet gelukt, met een "N" naast de HELIUScode.
- In beide gevallen kun je vanaf het *Home* screen de volgende deelnemer selecteren of invoeren voor het volgende onderzoek.

23. Einde meting

- Het gebruikte kapje niet weggoien. Leg het op de plastic mat in de lade van het bureau. Oude kapjes zullen aan het einde van de week door Luca desinfecteerd worden en in één van de papieren pakjes gehouden worden.
