# Supplementary figures and images for: Reproducibility of sublingual microcirculation parameters obtained from sidestream darkfield imaging
Source: PLoS One. 2019 Mar 14;14(3):e0213175. doi: 10.1371/journal.pone.0213175 (PMC6417651; doi:10.1371/journal.pone.0213175)

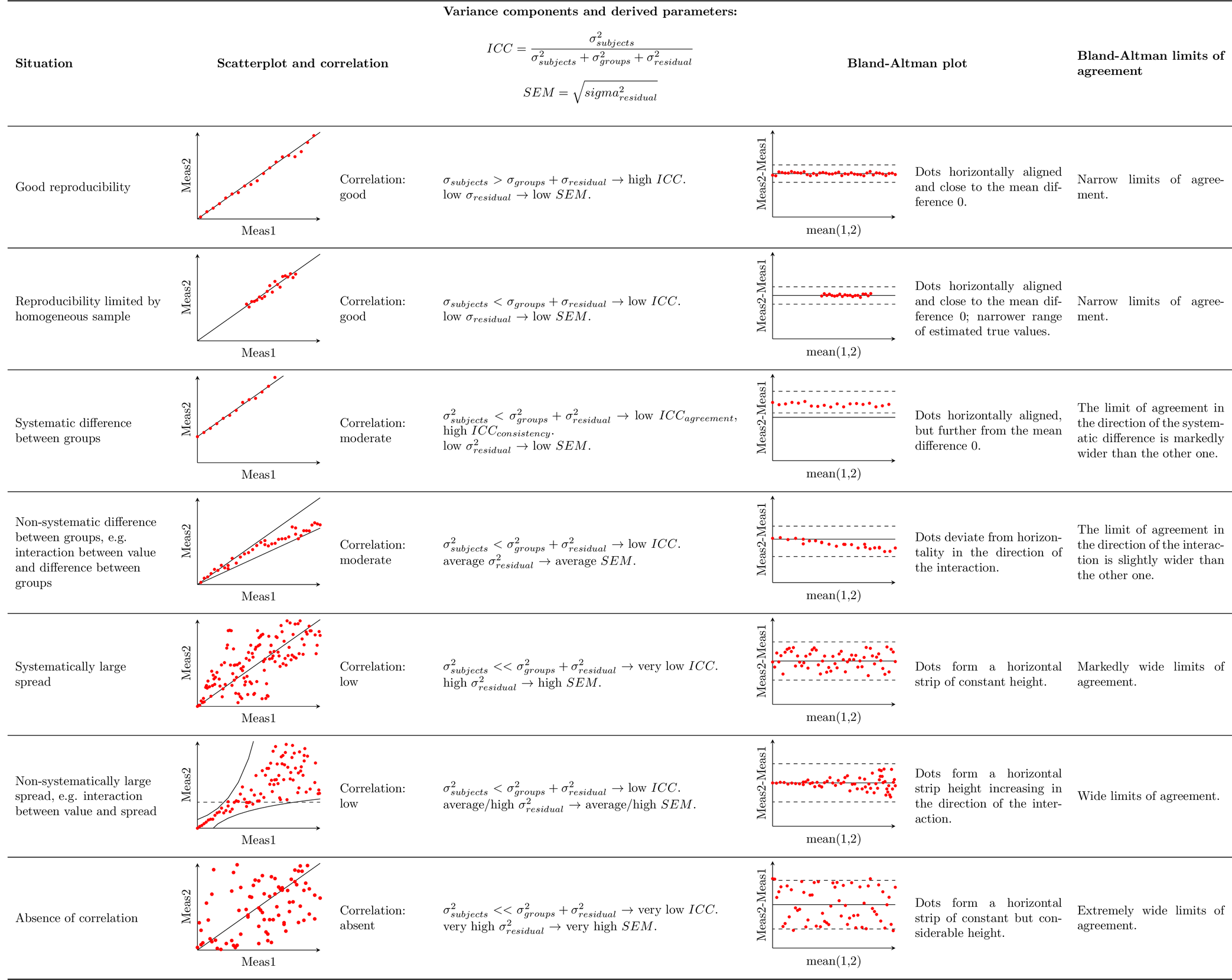

Supplement: S1 Fig — (TIF) [file pone.0213175.s005.tif]

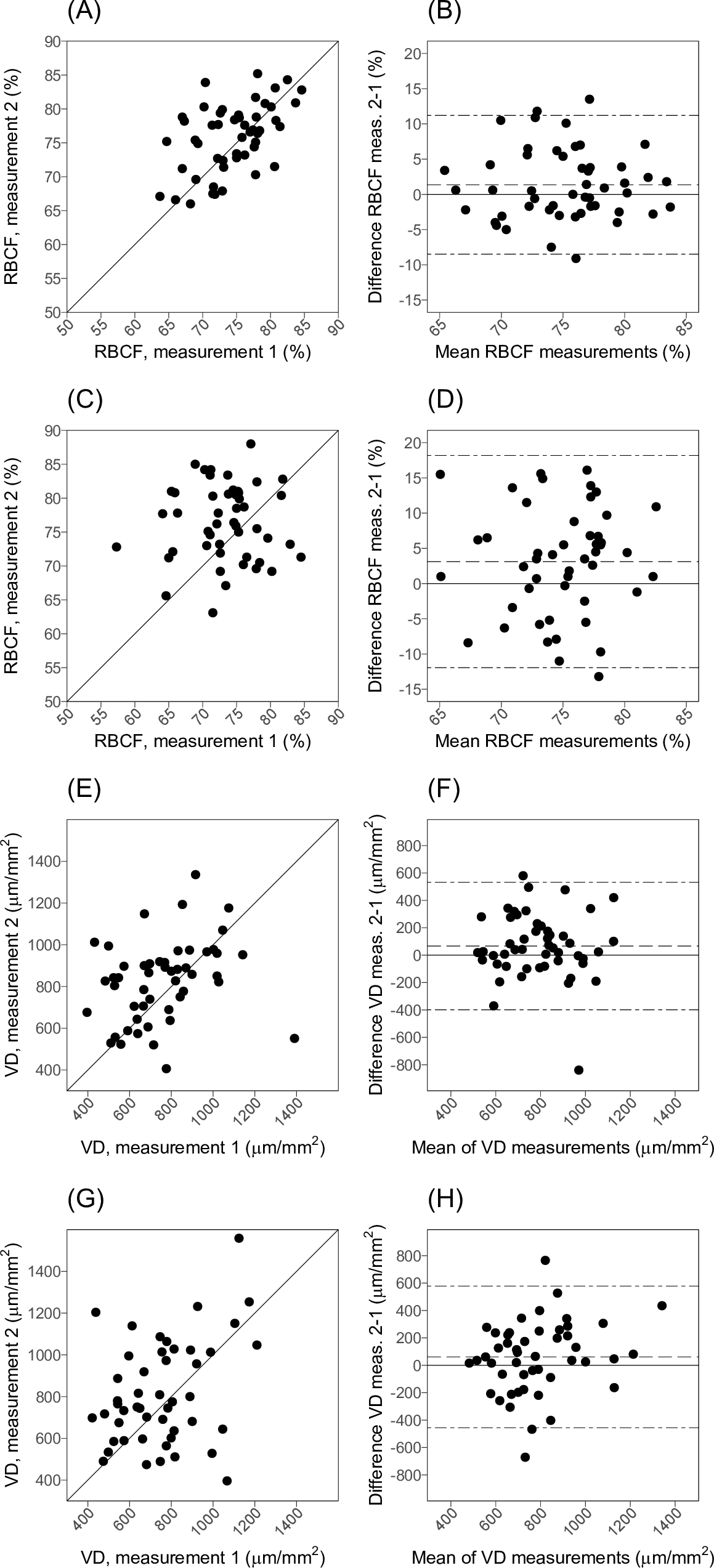

Supplement: S2 Fig — (A) Scatter plot of the intra-rater RBCF data. (B) Bland-Altman plot of the inter-rater RBCF data. (C) Scatter plot of the inter-rater RBCF data. (D) Bland-Altman plot of the inter-rater RBCF data. (E) Scatter plot of the intra-rater VD data. (F) Bland-Altman plot of the inter-rater VD data. (G) Scatter plot of the inter-rater VD data. (H) Bland-Altman plot of the inter-rater VD data. (TIF) [file pone.0213175.s006.tif]
